# Cumulative thermal stress predicts functional change in coral reef fish communities

**DOI:** 10.1098/rspb.2026.1955

**Published:** 2026-10-28

**Authors:** Anthony C. Markey, Damien A. Fordham, Rick D. Stuart-Smith, Graham J. Edgar, Christopher J. Brown, Camille Mellin

**Affiliations:** ^1^ The Environment Institute and School of Biological Sciences, Adelaide University, Adelaide, South Australia, Australia; ^2^ Centre for Macroecology, Evolution and Climate, GLOBE Institute, University of Copenhagen, Copenhagen, Capital Region of Denmark, Denmark; ^3^ Centre for Global Mountain Biodiversity, GLOBE Institute, University of Copenhagen, Copenhagen, Capital Region of Denmark, Denmark; ^4^ Institute for Marine and Antarctic Studies, University of Tasmania, Hobart, Tasmania, Australia; ^5^ Aquatic Sciences, South Australian Research and Development Institute, Adelaide, South Australia, Australia

**Keywords:** marine heatwave, tropical fish ecology, community ecology, ocean warming, functional traits, trait ecology

## Abstract

Marine heatwaves are altering coral reef fish communities through a combination of direct impacts of thermal stress and indirect impacts that degrade coral habitats. Impacts may vary for species in different functional groups (e.g. herbivore replacement of corallivores), altering the functional composition of fish communities that is critical to reef resilience. However, our understanding of trait-based responses of fish communities to marine heatwaves is inadequate, and mostly inferred from local scales and low numbers of species. Here, we use a Bayesian joint species distribution model based on data for 500 fish species from 286 sites surveyed across northern Australia to evaluate the impacts of marine heatwaves on species probability of occurrence and their interactions with four functional traits. The effects of degree heating weeks and coral loss on individual species probability of occurrence had the highest total and proportionally negative responses, respectively. Thermal affinity's positive response to cumulative heat stress (i.e. degree heating weeks) was the strongest trait effect. Community-level analyses predicted losses in habitat specialists, large-bodied fishes, corallivores and pelagic feeders along a gradient of marine heatwave stress. The loss and restructuring of functional diversity suggest that heatwaves will continue eroding fish-dependent ecological roles and associated ecosystem services.

## 1. Introduction

Anthropogenic disturbances and environmental changes are provoking shifts in ecological communities across all biomes [1], driving the formation of novel communities that are often dominated by adaptive, generalist species [2,3] and degrading crucial ecosystem services [4,5]. Of particular recent concern are acute disturbances, including extreme weather events, which can rapidly alter community composition and reduce critical ecological functions [6]. Functional diversity losses from extreme events pose risks to community recovery and can result in alternative ecosystem states with diminished ecological functions [7,8].

Among all marine ecosystems, coral reefs have the highest species and functional richness (i.e. the range of different functional groups within a community) [9,10], providing important services to nature and people including economic (fisheries and tourism), health-related (nutrition), environmental (coastal protection) and cultural benefits [5]. Despite their distinct importance, coral reefs are some of the most threatened ecosystems in the world, suffering from an array of chronic (e.g. overfishing, pollution and global warming) and acute disturbances (e.g. tropical cyclones and marine heatwaves) that erode diversity, jeopardizing their ability to resist and recover from future, subsequent or repeat disturbances [11–13]. Resilience to these stressors, many of which are human-driven, generally varies widely among reefs depending on community composition, disturbance history and local management [14,15].

Thermal stress caused by prolonged heat during increasingly frequent and severe marine heatwaves is now the primary threat to the persistence of Earth's coral reefs [16]. Heat stress causes corals to expel their symbiotic algae and bleach [17]. Although other climatic and environmental stresses can also cause or exacerbate bleaching, severe and long-lasting heatwaves often result in mass bleaching and subsequent coral mortality [16]. Spatial and temporal patterns of future coral bleaching based on global daily projections of heatwaves and associated heat stress suggest that maximum heat stress experienced by coral reefs of the Great Barrier Reef in Australia, as measured by degree heating weeks, will double by 2050 under an SSP5-8.5 climate scenario [18].

Marine heatwaves can affect tropical reef fish communities through direct mechanisms (e.g. metabolic changes, extirpation, reduced fitness and increased mortality rates) as well as indirect mechanisms (e.g. degradation of coral habitat or altered species interactions) [19]. Fish species vary greatly in their response to thermal stress and coral habitat loss, depending on their reliance on corals for food and shelter [20,21] and their proximity to thermal limits [22]. It follows that some functional groups are more vulnerable than others, based on traits such as diet, habitat specialization or thermal affinity. This reshuffling of tropical reef fish species composition owing to amplified marine heatwaves is reshaping fish functional trait assemblages and trait group diversity [23–25]. These altered communities are increasingly dominated by small-bodied herbivores at the expense of specialist species, including corallivores, further limiting coral reef ecosystem resistance to disturbance [21,26,27]. Cumulative disturbances, with little recovery time between them, are causing loss of specialist species and functional homogenization in coral reef fish communities [25,26]. Consequently, understanding which functional traits are most affected by marine heatwaves is crucial for understanding and minimizing further loss of reef fish biodiversity.

Here we use a joint species distribution modelling framework, namely Hierarchical Modelling of Species Communities (HMSC) [28], to (*i*) identify the coral reef fish species that are most likely to ‘win’ and ‘lose’ from severe marine heatwaves in tropical Northern Australia, (*ii*) establish the extent to which functional traits can help predict fish response to thermal stress and associated habitat loss and (*iii*) predict proportional functional composition changes along a gradient of marine heatwave severity. Our joint species distribution model incorporates species occurrence data, environmental variables, species traits and latent species co-occurrence patterns, allowing us to capture the drivers of fish community structure. We assess whether communities with distinct proportional trait group representation (hereafter ‘functionally distinct communities’) are disproportionately at risk of decline, potentially threatening fish functional richness and diversity as marine heatwaves increase in severity.

## 2. Materials and methods

### (a) Overview

We used standardized underwater visual census (UVC) surveys of tropical reef fish communities in Northern Australia from the Reef Life Survey (RLS) [29] to build HMSC [28]. HMSC's ability to incorporate species functional traits, alongside environmental variables, allowed the prediction of fish community functional change along gradients of thermal stress and coral cover. We clustered species into communities characterized by similar functional trait composition (hereafter ‘functional communities’), then we used HMSC to investigate the combined impact of thermal stress and coral loss on these fish communities. A conceptual flow chart of the analytical steps followed to predict fish community functional change along a gradient of heatwave severity is presented in figure S1 in the supplementary material. All data and code required to reproduce the following analysis and results can be found at Mendeley Data [30].

### (b) Occurrence data

The RLS data were obtained from the Australian Ocean Data Network (AODN) online portal at https://portal.aodn.org.au/ [29,31]. Our dataset consisted of 1260 transects surveyed across 329 sites from 2010 to 2019, covering six ecoregions: central and southern GBR (*n* = 28 sites), Torres Straight northern GBR (54), Coral Sea (100), Gulf of Papua (5), Bonaparte Coast (19), and Exmouth to Broome (123; Bonaparte Coast and Exmouth to Broome collectively referred to as the North West Shelf). A total of 290 sites were surveyed prior to a major marine heatwave in 2016 (2010–2015) and 321 sites were surveyed post-heatwave (2016–2019), including 282 sites resurveyed before and after the heatwave. Sites where other disturbances occurred between the pre- and post-heatwave surveys (e.g. Tropical Cyclone Olwyn in 2015; Tropical Cyclone Debbie in 2017; Tropical Cyclone Owen in 2018) were excluded from the analysis. The impact of other disturbances between the pre- and post-heatwave surveys at the retained sites is likely to be negligible, as demonstrated elsewhere [32].

Fish occurrence data were collected by two divers following a 50 m transect and recording all fish species observed within a 5 × 5 × 50 m block on either side of the transect line. Cryptic fish species were then recorded by divers swimming near the seabed, extending 1 m from the transect line. These two transect survey methods were then combined to provide complete fish presence data for each site. Typically, two parallel transects at different depths were surveyed at each site (mean = 1.9 transects per site), with depths ranging from 1–25 m. Per cent live coral cover was ascertained from 20 photo-quadrats per transect, taken every 2.5 m (mean area approx. 0.4 × 0.3 m). Photo-quadrats were later scored by identifying functional groups under 5 points per photo, giving 100 total points per transect.

### (c) Environmental and fish trait data

Heat stress was measured using annual maximum degree heating weeks (DHW; °C-week) and annual maximum sea surface temperature anomaly (SSTa; °C) (table 1). DHW is the most widely used predictor for coral bleaching risk and is measured as cumulative positive temperature anomalies above the long-term summer maximum over a span of 12 weeks [41]. However, DHW does not differentiate between sustained, low-level heat stress and brief, intense heat episodes, both of which can elicit diverse coral responses in terms of bleaching and subsequent mortality (e.g. [42]). To address this issue, we used SSTa to differentiate brief, intense heat episodes. For surveys sampled pre-heatwave, the annual maximum DHW and SSTa at each site in the year the survey was conducted were extracted from the National Oceanographic and Atmospheric Administration Coral Reef Watch v3.1 daily global 5 km satellite composite product [33], while for surveys sampled post-heatwave, the 2016 values at each site were used. This enabled localized instances of heat stress prior to the 2016 marine heatwave to be accounted for while also quantifying the major disturbance impact of the 2016 marine heatwave, noting that no other significant heatwave or major disturbance occurred between 2016 and the post-heatwave surveys [32]. Correlations between SSTa-DHW, SSTa-live coral cover and DHW-live coral cover were low to moderate yet all significant (Spearman *ρ* values = 0.439, 0.173 and –0.133, respectively; all *P* < 0.001).

**Table 1.** Summary of the variables used to fit the HMSC joint species distribution model.

variable category	variable name	mean ± s.d. [min–max]	rationale	source
**environmental covariates**	maximum degree heating weeks (maxDHW; °C-week)	5.50 ± 4.77 [0–17.82]	main proxy for coral bleaching severity during the 2016 marine heatwave	NOAA Coral Reef Watch [33]
	maximum sea surface temperature anomaly (maxSSTa; °C)	2.15 ± 0.49 [0.84–3.71]	indicates marine heatwave peak thermal stress	NOAA Coral Reef Watch [33]
	live coral cover (%)	21.27 ± 17.87 [0–100]	important indicator of reef health and function	Reef Life Survey Foundation [29]
	reef area within a 15 km radius (km^2^)	85.16 ± 85.86 [12.75–539.25]	reef connectivity suggested to influence reef resilience [34]	[35]
	land area within a 50-km radius (km^2^)	165.69 ± 486.86 [0-3308.99]	index of human influence and terrestrial pollution impact [13,15]	[35]
	wave energy (Mean kW m^–1^)	12.13 ± 5.55 [0.024–22.66]	shapes fish assemblages at local to regional scales [36]	[35]
**fish species traits**	species generalization index (SGI; a measure of habitat niche breadth)	12.43 ± 7.83 [0.26–37.79]	habitat specialists are disproportionately impacted by coral bleaching [25]	[25]
	trophic group	corallivore (*n* = 28); herbivore (*n* = 151); benthic invertivore (*n* = 196); planktivore (*n* = 69); higher carnivore (*n* = 56)	trophic groups respond differently to marine heatwaves and influence reef resilience [26,27,37]	[38]
	maximum length (cm)	32.77 ± 34.59 [1.7–300]	smaller species expected to be more negatively impacted by structural complexity loss [27]	[38]
	thermal midpoint of species range (°C)	26.2 ± 1.3 [21.9–29.8]	cool-affinity species expected to be more negatively impacted by thermal stress than warm-affinity species [39,40]	[39]

To account for other reef processes that shape fish communities [34,36], additional information on biophysical conditions (wave energy with corrections for sheltered coastlines) and seascape context (coral reef and land cover within a 15 and 50 km radius, respectively) was sourced from the Marine Socio-Environmental Covariates (MSEC) dataset for the world's oceans [35] (table 1). After excluding sites with missing environmental information, our dataset contained 1062 surveys at 286 sites. Of these, 274 and 273 were surveyed before and after the 2016 marine heatwave, respectively, with 261 sites resurveyed.

Four fish species traits that are likely to influence species responses to marine heatwaves were used to quantify trait-based responses to heat stress (table 1). These were (*i*) maximum length (cm) [38]; (*ii*) thermal niche midpoint (°C; a standardized and continuous measure that approximates the sea temperatures at which species’ local abundances, and therefore ecological success, are greatest based on field observations across each species's entire distribution [39]); (*iii*) trophic group [38] and (*iv*) species generalization index (SGI; a measure of habitat niche breadth with lower values indicating more specialized species [25]). Out of a total of 1046 species observed across all sites, 705 species had trait information available. Since our modelling framework cannot handle NA values, only species with trait information were retained. We tested for collinearity between traits for the 705 species for which trait information was available. Correlations between maximum length-thermal niche midpoint and SGI-maximum length were non-significant (Spearman *ρ* = –0.046 and 0.069, respectively; *P*-value > 0.05), while the correlation between SGI and thermal niche midpoint was moderately negative (Spearman *ρ* = –0.255; *P*-value < 0.0001).

### (d) Hierarchical modelling of species communities

We used HMSC to evaluate species- and trait-based responses to the 2016 marine heatwave [28]. This joint species distribution model predicts the probability of species occurrence based on environmental variables, traits and species co-occurrences (summarized in table 1). HMSC enables simultaneous modelling of species probability of occurrence and residual species co-occurrence for community-level predictions. To reduce zero-inflation and ensure model convergence [43] and computational efficiency, we restricted our analysis to the 500 most common species (out of a total of 705 species) across pre- and post-heatwave surveys (figure 1; supplementary material, table S1). Mean survey prevalence ± s.d. [min – max] of the 500 selected species was 0.11 ± 0.13 [0.01 – 0.83]. Permutational multivariate analysis of variance and dispersion tests, with the R package ‘vegan’ [44], showed that only 0.8% of trait variation was explained by the two groups of species (all 705 species with trait data versus 500 selected species) and that species mean distance to group centroids in trait space only differed by 10.7%, suggesting a negligible functional shift between groups. Kolmogorov-Smirnov (numeric traits) and chi-square (trophic group) tests were used to test for changes in the distribution of traits between the full set of species (*N* = 705) and the subset of species retained for the analysis (*N* = 500) using the ‘dgof’ R package [45] (supplementary material, figure S2).

**Figure 1.**
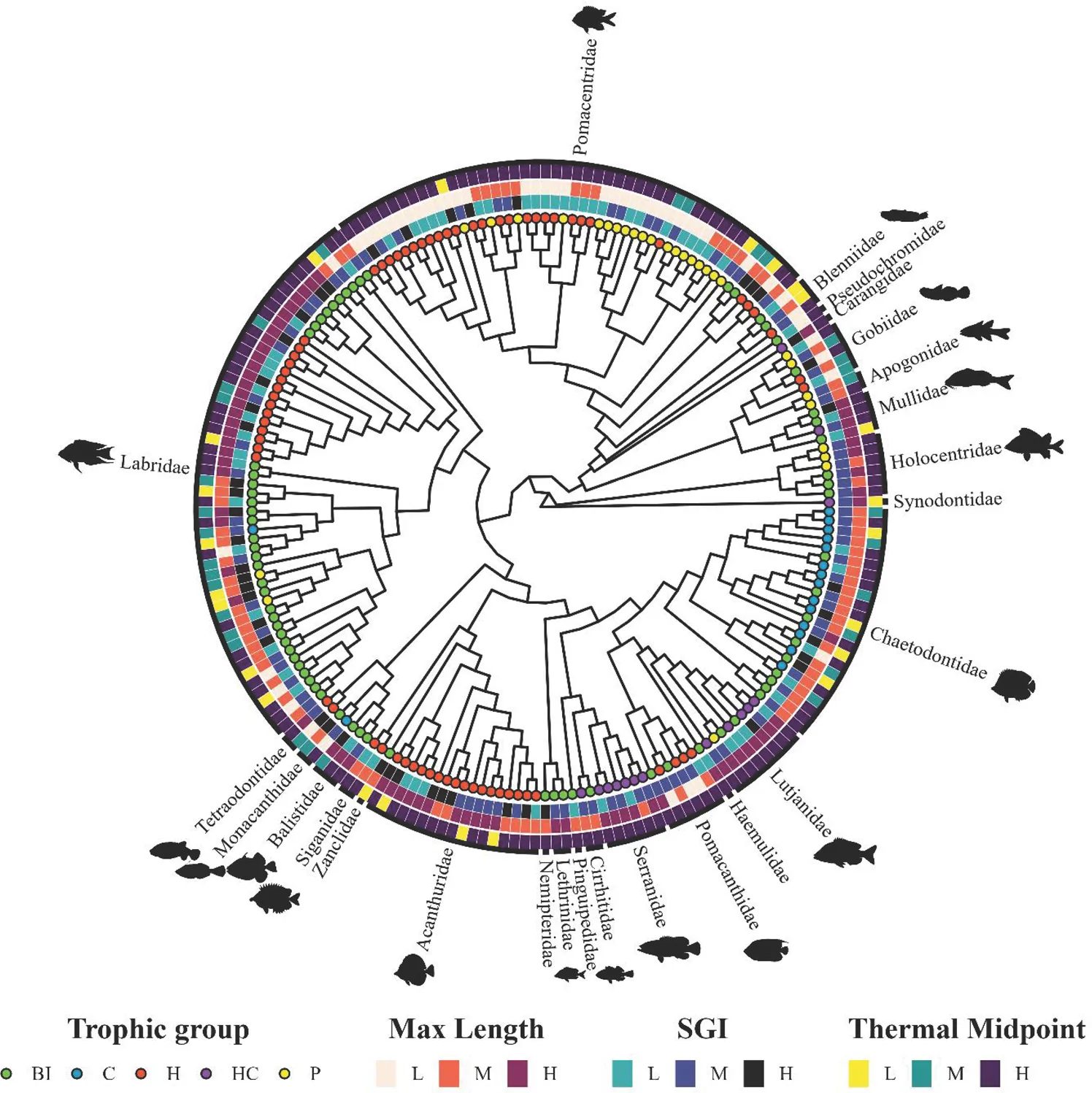
Circular phylogram heatmap of species trait categorizations used in the functional clustering of fish communities of the 200 most prevalent species modelled by HMSC. Tip points are coloured by trophic group: (i) BI = benthic invertivore (green), (ii) C = corallivore (blue), (iii) H = herbivore (red), (iv) HC = higher carnivore (purple), and (v) P = planktivore (yellow). The concentric rings show the groupings of species trait values into low (L), medium (M) and high (H) categories for the species generalization index (SGI), maximum length and thermal midpoint, outwardly. Black arches group families and are labelled, with silhouettes showing generic family form for families with two or more species represented.

We fit the HMSC binomial model with a probit link function to the species occurrence data using the ‘Hmsc’ package in R [43,46]. We included two latent random variables: (*i*) a site-level random variable to account for the non-independence of surveys conducted at the same site, and (*ii*) a pre- versus post-heatwave random variable to account for the repeated nature of the surveys. HMSC is a Bayesian modelling technique that uses Monte Carlo Markov chain (MCMC) sampling to estimate posterior distributions of model parameters. We used the default priors suggested by Ovaskainen *et al.* [28]. We sampled four MCMC chains, each with a total of 350 000 samples consisting of 200 000 burn-in samples, then retaining every 300th sample, giving a retained total of 500 for each chain. We assessed model convergence using post scale reduction factors (PSRFs; <1.1) for three parameters: β (species-environment), Γ (trait-environment), and Ω (species co-occurrence). We evaluated model goodness-of-fit using Tjur-R^2^ [47] and the area under the curve (AUC).

We used a 10-fold cross-validation procedure to evaluate the predictive power of the model. This process involved randomly partitioning sites into 10 groups and fitting ten HMSC models, each excluding one group of sites as validation data. Each site partition is used as validation data once, ensuring information from each site is used during the cross-validation process. We evaluated model predictive performance using Tjur-R^2^ and AUC calculated from the validation data.

Within the HMSC framework, species’ responses to environmental variables (i.e. anomalous thermal conditions or change in coral cover) are represented by the parameter β, which estimates the change in a species’ probability of occurrence along an environmental gradient. For example, a species’ β estimate for DHW thus quantifies, across all the surveys in the training data, the association between species distribution and the magnitude of cumulative thermal stress experienced at those sites. Likewise, trait associations with environmental variables, represented by the parameter Γ, thus quantify, across all the surveys in the training dataset, the association between the occurrence of species with certain trait values and the environmental conditions observed at those sites. We used these parameters to distinguish the ‘winners’ and ‘losers’ of the three primary marine heatwave-related environmental variables (maxDHW, maxSSTa and live coral cover). To preserve consistency of response direction for ‘winners’ and ‘losers’, coral cover responses were negated and hereafter referred to as responses to coral loss. For Γ, effects whose 80, 90 and 95% credible intervals excluded zero were interpreted as providing low, moderate and high evidence for a non-zero effect, approximately corresponding to probabilities of direction of 0.90, 0.95 and 0.975, respectively. As HMSC uses dummy variables for categorical variables, trophic group Γ responses are relative to a reference group incorporated into intercept parameters—leaving the reference group Γ estimates impossible to measure directly. To account for this, a sensitivity analysis using three models with different reference groups was fitted to represent the bottom (herbivore), middle (benthic invertivore) and top (higher carnivore) trophic levels. Hereafter, unless stated otherwise, analyses and results are based on the benthic invertivore reference group model because this was deemed to best represent differences between higher and lower trophic groups.

Species overall response to marine heatwaves was measured as the difference in predicted probability of occurrence (calculated as the difference between pairwise samples of each MCMC chain prediction) between a low-impact state (SSTa = 0°C, DHW = 0°C week^–1^, live coral cover = 100%) and a median heatwave state (median values of post-heatwave data used in this analysis: SSTa = 2.31°C, DHW = 7.62°C week^–1^ and live coral cover = 15%). We chose to limit this prediction to median rather than maximum levels of heatwave exposure, as prediction uncertainty is lowest around the median.

### (e) Functional clustering of pre-heatwave fish communities

We clustered sites by the compositions of species traits prior to the 2016 marine heatwave to identify functionally similar communities. We then used the HMSC model to predict changes in the functional communities along a marine heatwave gradient (supplementary material, figure S1). We excluded sites where only a single transect was surveyed (20.1% of sites). For sites surveyed in multiple years pre-2016 (*n* = 1 site), we selected the earliest year, while for sites with more than two surveys in a single year (8.0% of sites), we randomly selected two surveys from that year. We subsequently aggregated this standardized pre-heatwave species occurrence matrix from transect- to site-level. If a species appeared on at least one transection at a particular site, we recorded it as present.

We grouped continuous species traits into low, medium and high categories (figure 1; supplementary material, table S2), resulting in a binary species-by-trait categorical matrix (i.e. a herbivorous, high thermal affinity species would have a value of 1 in the herbivore column, 0 in all other trophic group columns and values of 0, 0 and 1 for low, medium and high thermal midpoint, respectively). The categories for maximum length and thermal midpoint were selected to represent ecologically relevant breaks [48,49], while SGI was split according to its geographical distribution [25]. We then multiplied the pre-heatwave presence-absence matrix by the species-by-trait matrix, producing a site-by-trait richness matrix. This site-by-trait richness matrix was standardized by dividing by the total number of species present at each site, thus reflecting proportional trait richness. This final matrix thus contained the proportion of species from each trait present at a given site prior to the heatwave (supplementary material, figure S1). For example, if a site had 10 species present, including three species with low SGI, five species with medium SGI and two species with high SGI, their proportional values would be 0.3, 0.5 and 0.2, respectively.

We split this site-by-proportional trait richness matrix into functionally distinct clusters using k-means clustering. The optimal number of clusters was determined using the Calinski–Harabasz criterion [50], choosing from a range of 3–8 clusters with the ‘cascadeKM’ function from the ‘vegan’ R package [44]. Non-metric multidimensional scaling (NMDS) in three dimensions facilitated visualization of these clusters in functional space. We identified indicator trait categories associated with each cluster using the indicator index ‘indval’ from the ‘labdsv’ package [51].

### (f) Community predictions along environmental gradients

We used the HMSC model to predict species probabilities of occurrence at five quantile intervals along a marine heatwave gradient, from the lowest intensity observed post-heatwave (DHW = 0°C week^–1^, SSTa = 1.63 °C, live coral cover = 90%) to the highest observed intensity values (DHW = 16.74°C week^–1^, SSTa = 3.71 °C, live coral cover = 0%; supplementary material, figure S1). For each interval of heatwave severity, we multiplied predicted species probability of occurrences with the pre-heatwave presence-absence matrix, resulting in a species probability of occurrence conditional on pre-heatwave presence for each interval (i.e. five matrices).

These matrices were then multiplied with the binary trait matrix using standard matrix multiplication, and standardized using the method described above to represent proportional trait richness. We used NMDS for each functionally distinct community previously determined to visualize community functional change along environmental gradients in two dimensions. We measured changes in the proportional probability of occurrence of each trait bin as the change in Euclidean distance from a trait group score (i.e. species score in traditional NMDS) to the centroids of the lowest and highest intervals of the marine heatwave gradient (supplementary material, figure S1).

For example, if a trait group score has Euclidean distances of 0.1 and 0.05 from the centroids of the lowest and highest marine heatwave interval community predictions, respectively, then the change in community proportional probability of occurrence for that trait is 0.05.

## 3. Results

### (a) Model performance

Model evaluation showed high model convergence and moderate to good explanatory power. Mean post-scale reduction factor (PSRF) (max; % < 1.1) values for the three assessed parameters were: β 1.003 (1.18; 99.9%), Γ 1.002 (1.007; 100%), and Ω 1.014 (2.37; 98.72%; supplementary material, figure S3). Explanatory mean Tjur-R^2^ and area under the curve (AUC) ± s.d. [min – max] were 0.21 ± 0.13 [0 – 0.82] and 0.92 ± 0.066 [0.72 – 1], respectively. Predictive mean Tjur-R^2^ and AUC ± s.d. [min – max] from 10-fold cross-validation were 0.024 ± 0.038 [–0.036 – 0.30] and 0.64 ± 0.14 [0.24 – 0.98], respectively. The lower predictive R² and AUC values compared with their explanatory counterparts suggest that the model explains moderate variation in the data with good rank classification; however, its generalizability to new observations is limited.

### (b) Hierarchical modelling of species communities

Differences in predicted probability of occurrence between no-heatwave and median heatwave environmental conditions found high evidence that six species responded positively to heatwave conditions, while 31 responded negatively, moderate evidence for 10 additional ‘winners’ and 29 additional ‘losers’, and low evidence for an additional 13 and 45, respectively (figure 2; supplementary material, table S1). Our HMSC modelling showed high evidence that more species responded to degree heating weeks (DHW; *n* = 132) than to sea surface temperature anomaly (SSTa; *n* = 105) and coral loss (*n* = 85; supplementary material, figure S4 and table S1), indicating cumulative heat stress as a primary determinant of fish community composition. It also revealed that negative responses of fish to coral loss were proportionally more commonly observed (75.3% of 95% credible intervals not overlapping 0) compared with negative responses to SSTa (71.4%) or DHW (50%).

**Figure 2.**
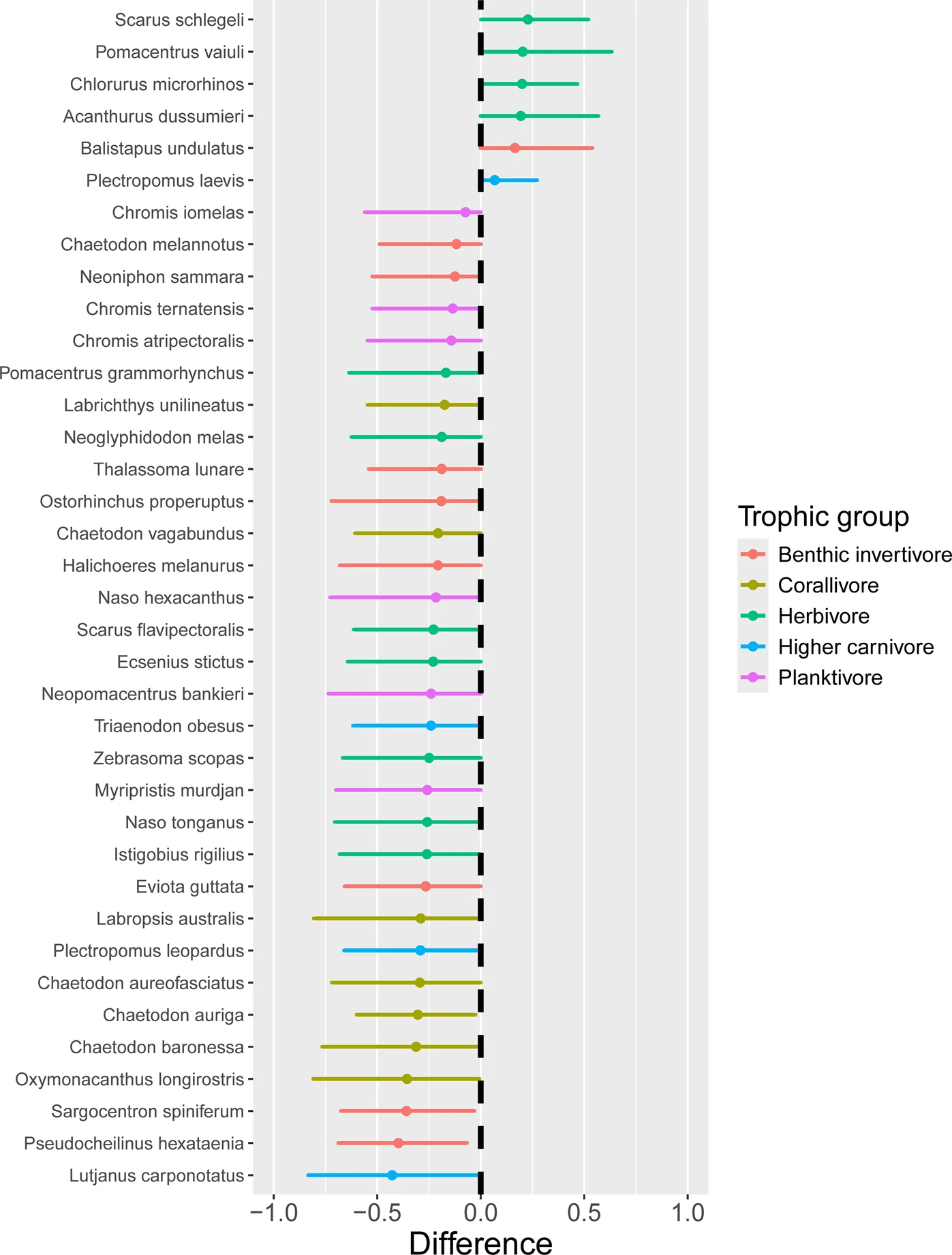
Forest plots of species predicted difference in probability of occurrence between no heatwave and median heatwave environmental conditions, with positive responses representing an increase in probability of occurrence in a median heatwave scenario. Error bars represent 95% credible intervals. Points are coloured by trophic group. Only species with strong evidence for an effect are displayed (i.e. 95% credible intervals not overlapping 0).

Synchronicity was rare among species occurrence responses (positive and negative) to the marine heatwave variables. The highest synchronicity was for negative species responses to coral loss and cumulative heat stress responses (i.e. DHW), where 20 species responded in similar directions to both heatwave variables. For other combinations of variables, ≤7 species showed synchrony in the direction of their responses (table S3). Disparity between species responses to heatwave variables was slightly more pronounced, particularly between comparisons of DHW-SSTA, with 25 negative-positive and 38 positive-negative responses observed (table S3). No species had negative or positive responses to all three heatwave variables.

Maximum length negatively affected species responses to cumulative heat stress (DHW; moderate evidence), but positively influenced species responses to peak thermal intensity (SSTa; low evidence), meaning larger-bodied fishes were more likely to be negatively affected by cumulative heat stress but less vulnerable to short peak intensities (figure 3). A higher thermal affinity had a positive influence on species responses to DHW (high evidence), but a negative association with coral loss (low evidence). Species SGI had a negative influence on species responses to coral loss (low evidence) and DHW (high evidence), and a positive influence on species responses to SSTa (high evidence).

**Figure 3.**
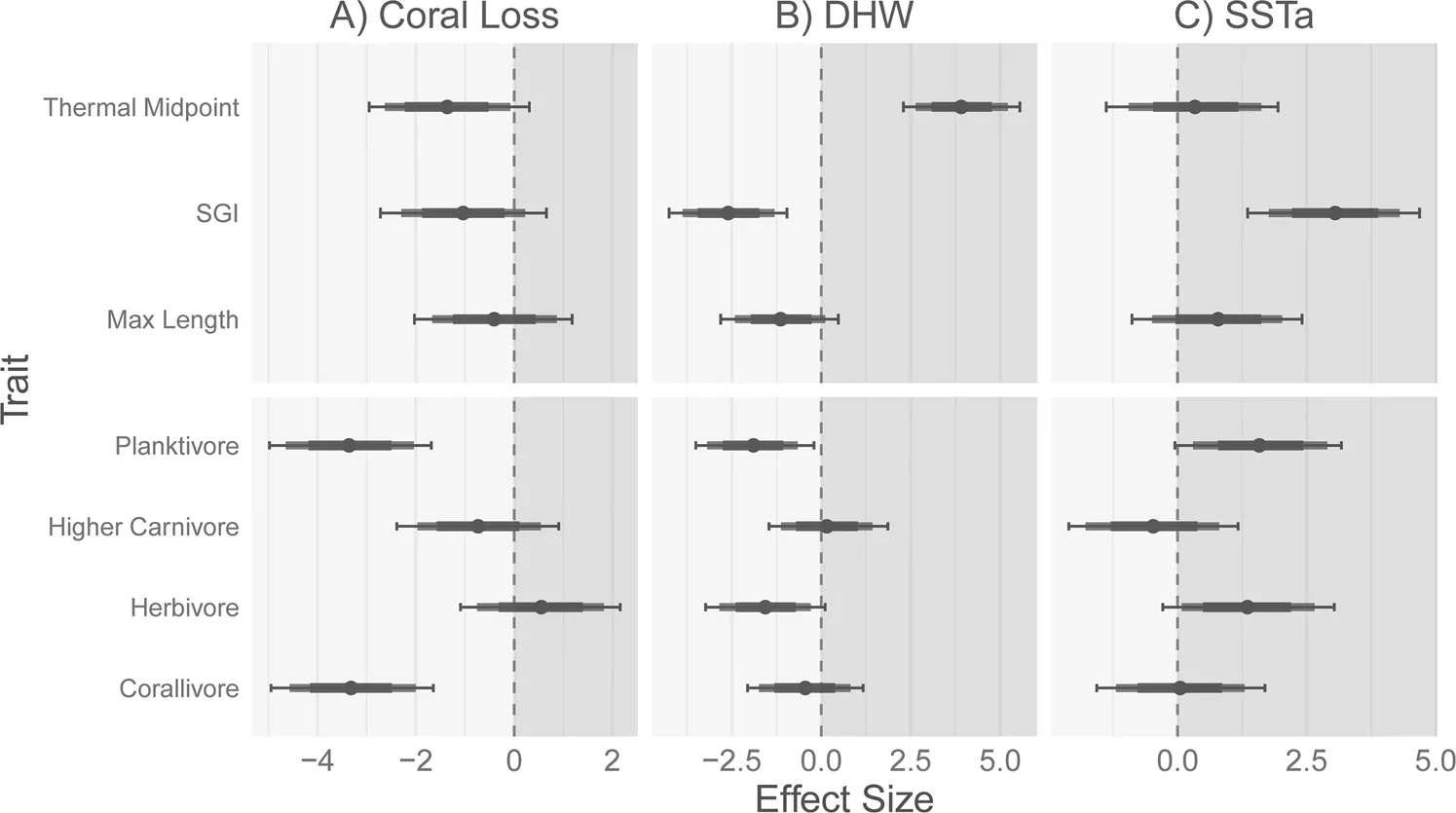
Forest plots of Γ (trait-environment) effect sizes for (A) coral loss, (B) degree heating weeks (DHW) and (C) sea surface temperature anomaly (SSTa), split by continuous traits (top) and trophic groups (bottom). Posterior distributions have been scaled by dividing by their standard deviations. Displayed intervals in order of increasing thickness are 95, 90 and 80% credible intervals. Trophic group effects are relative to the dummy variable reference group of benthic invertivores.

Planktivores and herbivores responded negatively to DHW (high and moderate evidence, respectively) and positively to SSTa (high and moderate evidence, respectively). This result suggests that, for both trophic groups, the negative impact of marine heatwaves was greatest after prolonged exposure to moderately higher temperatures (i.e. high DHW and low SSTa). Planktivores and corallivores also negatively responded to coral loss (both high evidence). The direction of these trophic group effects was consistent across models with different reference groups. Additionally, in the model with herbivores as the reference group, benthic invertivores responded positively and negatively to DHW and SSTa, respectively (both moderate evidence; supplementary material, figure S5).

### (c) Functional analysis

Out of the total of 220 sites surveyed prior to the heatwave and meeting our selection criteria, K-means clustering identified four distinct community types comprising 30, 47, 77 and 66 sites (i.e. communities 1–4, respectively; 52.79% variance explained). Community 1 mostly included southern sites of the GBR and Coral Sea, characterized by species of low to medium thermal midpoint, habitat generalists, invertivores and medium-sized species (hereafter Southern community; figure 4; supplementary material, figure S6). Community 2 consisted of northern outer reefs of the Coral Sea and north-west shelf (hereafter Outer community). These sites were dominated by carnivores, planktivores and large-bodied species with high thermal affinity. Community 3 included almost all sites on the northern GBR and most from the north-west shelf of the four groups (hereafter Northern community). These sites were characterized by small-bodied, herbivorous and highly specialized fishes. Community 4 predominantly included sites in the Coral Sea and was characterized by medium SGI as an indicator trait (hereafter Coral Sea community).

**Figure 4.**
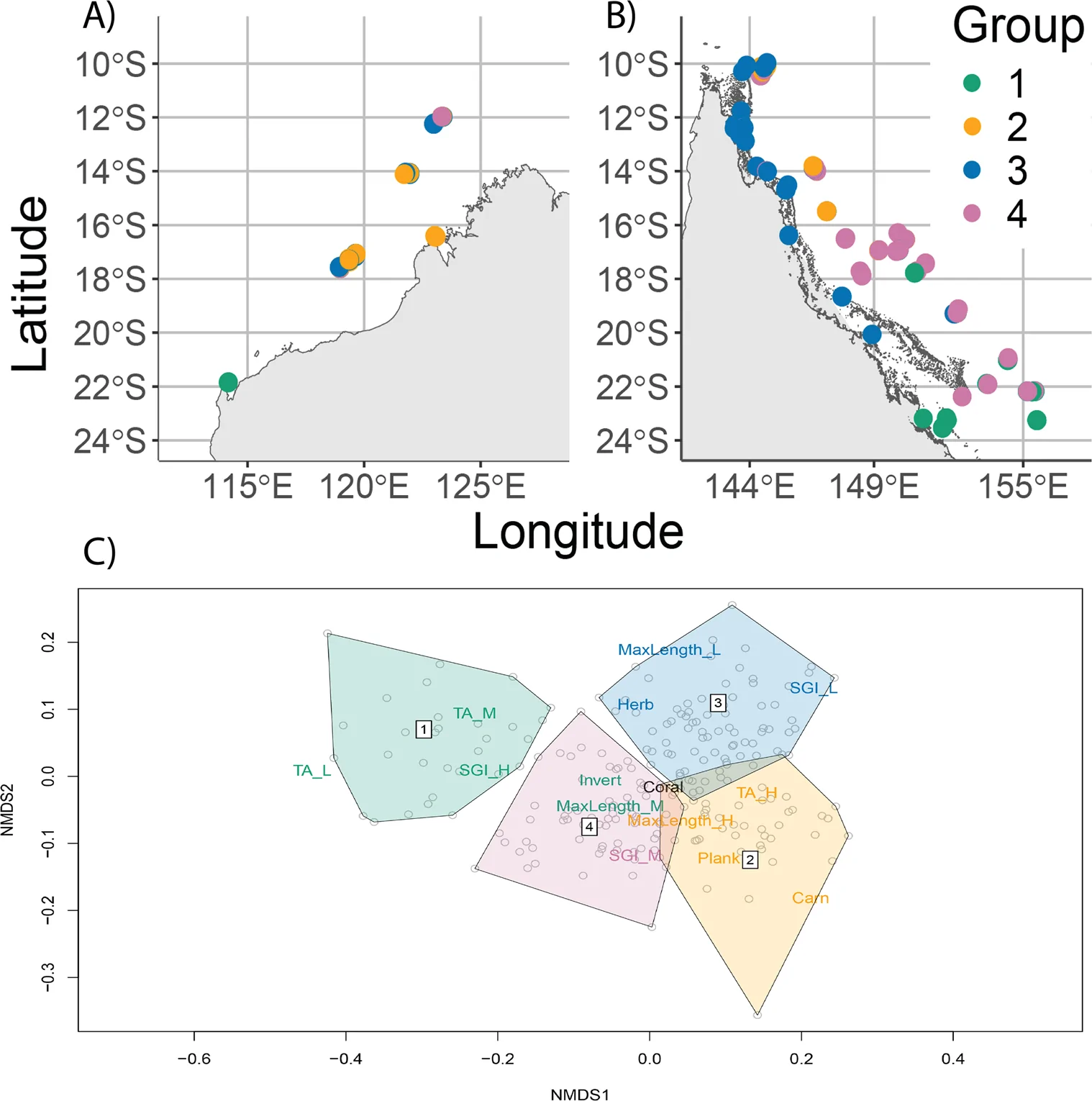
(A and B) Maps displaying the location of sites coloured by community grouping (1 = Southern community, 2 = Outer, 3 = Northern and 4 = Coral Sea). C) An NMDS bi-plot showing the position of each community grouping (coloured hulls) in functional space. Indicator traits are labelled and coloured by their respective community. Traits are labelled as TA = thermal affinity, SGI = species generalization index, MaxLength = maximum length, plank = planktivore, carn = higher carnivore, herb = herbivore, invert = invertivore and Coral = corallivore. Trait suffixes are L = low, M = medium and H = high.

Our community-level analysis predicted that pre-heatwave functional composition has little impact on the direction of change in proportional trait representation, with our four functionally distinct communities responding in the same direction (i.e. all four communities losing or gaining proportional trait representation) for 11 of 14 trait groups (figure 5; supplementary material, figure S7). The exceptions were for the Southern Community, which, compared with the three other communities, showed opposite directional responses for planktivores (positive), herbivores (negative) and species with small maximum lengths (negative; figure 5)—all of which were underrepresented in the Southern Community's pre-heatwave data (figure 4).

**Figure 5.**
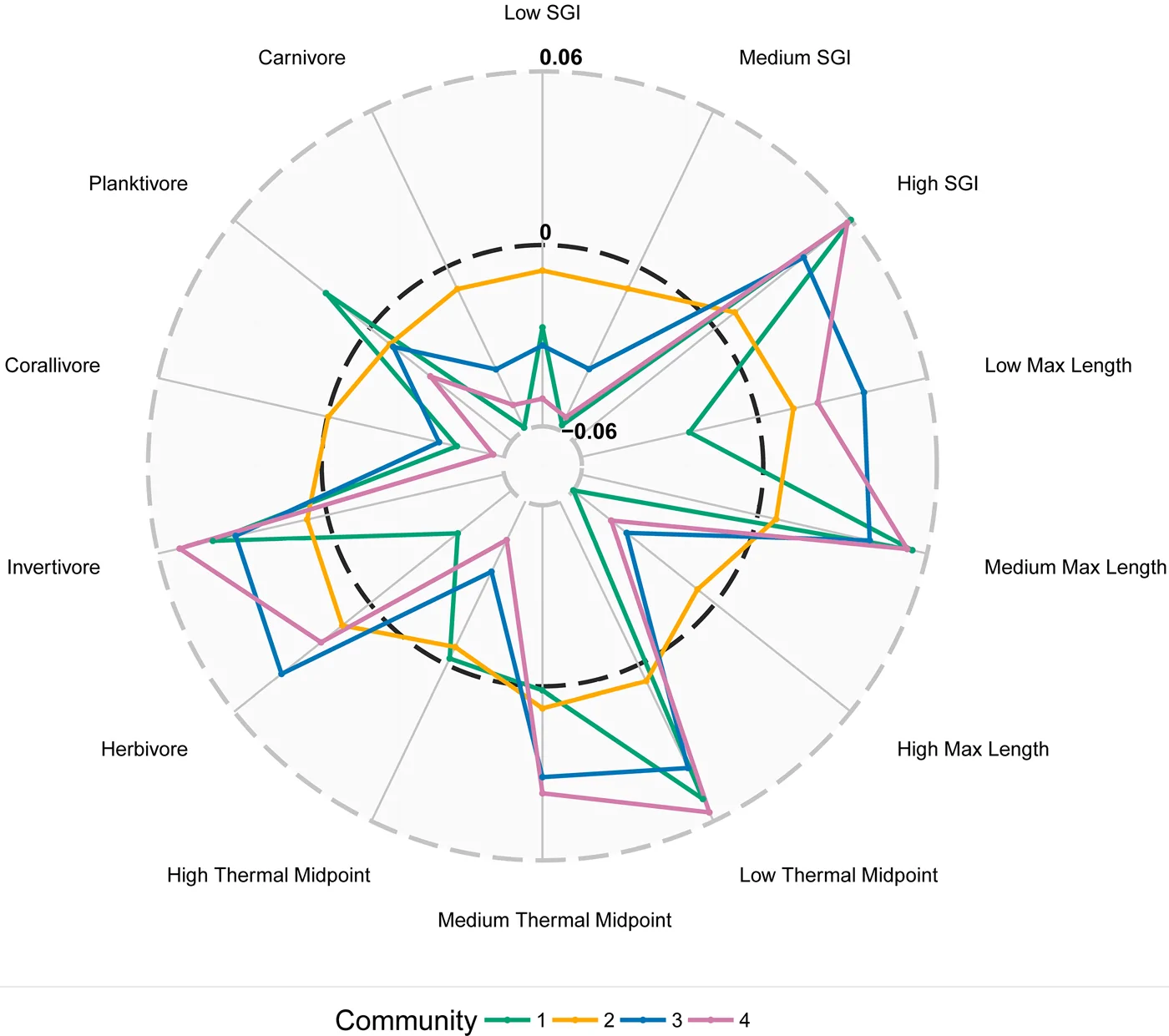
Predicted changes in the representation of functional groups within fish communities along a gradient of marine heatwave severity. Shown on the radial axis is the distance change in trait group NMDS centroids between the lowest and highest marine heatwave environmental gradient community predictions. A positive value means an increase in the proportion of species from a given trait group under severe heatwave conditions. Labelled radial axis values are the minimum and maximum distances observed across all communities and traits. Lines are coloured by fish communities (1 = Southern community, 2 = Outer, 3 = Northern and 4 = Coral Sea).

Our modelling predicted a gain in habitat generalists and a corresponding loss in low- to medium-SGI species across all communities along an increasing heatwave gradient (figure 5). Except for the Southern community, there was an overall predicted loss of large-bodied species and gains in small- to medium-sized fishes (figure 5). Proportional representation of high thermal affinity species was predicted to decline while lower thermal affinities were predicted to increase (figure 5). Among trophic groups, losses were predicted for higher carnivores, planktivores and corallivores, while invertivores and herbivores were predicted to increase along an increasing heatwave gradient (figure 5).

## 4. Discussion

Marine heatwaves are increasing in frequency and intensity, yet their impacts on fish communities remain poorly understood. This is partly owing to complex interactions among fish species and their habitats, and the diversity of their responses. Using a unique dataset of pre- and post-heatwave surveys of fish communities, we derived a joint species distribution model to identify the winners and losers of heat stress and subsequent coral loss and to quantify trait-based responses to the 2016 heatwave in tropical Australia. We show that thermal affinity was the primary trait associated with probability of occurrence along a heatwave gradient and identified six winners and 31 losers from a total of 500 species analysed. Notably, our metric of cumulative heat stress (DHW) had the most species with >95% posterior probability of an effect, although coral loss explained proportionally more negative responses—suggesting that cumulative heat stress acts as the main heatwave driver of species occurrence, but associated habitat loss is the most detrimental. Our community-level analysis and predictions showed that marine heatwaves consistently alter coral reef fish communities towards a dominance of small-bodied, generalist and herbivorous fish species. We highlight that functional diversity loss is occurring across all community types, possibly reducing the resilience of coral reef fish communities to future disturbances if recovery does not follow.

Among all the fish species traits that we considered, the interaction between thermal affinity and DHW had the largest influence on species occurrence, indicating that species with higher thermal affinities have better tolerance to cumulative thermal stress experienced during marine heatwaves. The patterns predicted by our modelling could reflect a correlation in the latitudinal distributions of thermal affinity and DHW [16,40], with greatest DHW values experienced in warmer regions where more species with higher thermal affinities occur. Therefore, the strong positive influence of thermal affinity on species probability of occurrence responses to DHW could reflect this bias. Furthermore, the results likely reflect that higher thermal affinity species may have more frequently occurred near the warmer edges of their thermal range prior to the heatwave, and consequently experienced greater losses at higher SST anomalies [39]. Our community-level analysis supports this, with the two lowest latitude communities predicted to experience the greatest loss in higher thermal affinity species, suggesting a latitudinal (i.e. thermal range) effect linked to previously observed processes such as poleward migrations leading to tropicalization, and corroborates previous findings of increased coral vulnerability at lower latitudes [13,52,53].

Responses related to the species generalization index (SGI) were also complex and impacted by its correlation with thermal affinity and DHW [25]. The negative effect of DHW on probability of occurrence for habitat generalists, in the species-level analysis (figure 3), may have occurred as a result of this correlation, being an outcome of the positive responses observed in species with higher thermal affinity (i.e. as warmer thermal affinity species tend to be more specialized and they had a positive response to increasing DHW, generalists were lost, simply owing to this correlation). The community-level predictions in figure 5 were perhaps more convincing of the relationships between SGIs and heatwave impacts where thermal stress and coral cover were set to be uniformly high and low, respectively. In these circumstances, the proportional richness of habitat generalists was clearly predicted to increase, alongside decreases in habitat specialists (figure 5). This prediction of ecological generalization in our coral reef fish communities follows previous research highlighting the loss of specialized fish species following heatwave impacts [24,25]. It also aligns with rare and specialized species contributing the most to critical ecosystem functions [54], meaning future increased marine heatwave frequency and severity poses a threat to continued homogenization of reef fish communities and their ability to provide valuable ecosystem services [5,23]. However, it is important to note that the species selection method employed in this study excluded the rarest 29% of species that also tend to be specialists (supplementary material, figure S1), so the shift towards generalist species may be even greater than predicted.

Our trait-based responses suggest evidence for trophic restructuring from marine heatwaves, namely negative responses of planktivores and corallivores to coral cover loss (strong evidence). Fish communities in the Seychelles dominated by corallivores and planktivores have been shown to be more sensitive to coral bleaching events; however, overall trait redundancy increased resistance to changes in trait structure [26]. As such, a predicted loss of corallivores and planktivores from MHWs is expected but, if unchecked and a loss of redundancy eventually occurs, could erode community resilience to future disturbances. It is worth noting, however, that our model predictions relate to a single extreme event, and the temporal persistence of predicted reductions remains unknown, and recoveries may occur.

Concurrently with reductions in corallivores under increased habitat loss or erosion, herbivorous fishes responded more positively to coral loss compared with all other trophic groups. Herbivores had gains in proportional representation in all but the southern communities in our community-level analyses. While high herbivore species richness and redundancy can reduce macroalgal abundance on coral reefs and promote coral growth [37], different guilds of herbivores can have varying impacts on coral communities [55,56]. We categorized herbivore guilds into a single group because model convergence was compromised with an increased number of groups. Despite this, based on β responses, cropping guilds of herbivores such as algal farmers and browsers had a 14% positive probability of occurrence response to DHW, while their scraping counterparts had zero. The loss of excavating and scraping herbivores can facilitate phase shifts to algal-dominated reefs, as they are integral in maintaining a healthy coral state [55]. Furthermore, the bite process leaving exposed calcium carbonate by scrapers promotes coral recruitment compared with cropping bites by algal farmers promoting turf growth [56,57]. Alternatively, though, four out of six scraping herbivore species responses (mostly parrotfishes; 95% credible) to coral loss were positive, which corroborates previous evidence of increased parrotfish abundance post-bleaching, potentially from enhanced nutrient availability from successional photoautotrophic microbes [58,59]. However, these species-level responses do not provide guild-level information, as individual species may exhibit other characteristics that influence their response to thermal stress and habitat loss. As such, future modelling studies could explore differential responses across multiple herbivore guilds, pending further innovations in model development and perhaps additional data availability.

A pattern of opposing responses to different stress elements from marine heatwaves (i.e. cumulative versus peak intensity) was observed in species-level responses and trait effects. Many species responded differently to heatwave duration and intensity, while very few responded similarly. Disparity in response direction for our thermal variables was also observed for SGI, planktivores, herbivores and benthic invertivores (model with herbivores as the reference group; supplementary material, figure S4). This has implications for variable selection in future marine heatwave research, as selection of a single metric could lead to widely different results and inferences.

Critical functional responses may have been missed owing to the limited number of functional traits modelled in our study for computational tractability. While we selected four traits previously suggested to influence fish species responses to marine heatwaves, many other traits that contribute to shaping coral reef fish communities (e.g. home range size, mobility and life history strategy) would warrant further investigation. Likewise, additional environmental variables not included in the analysis (e.g. primary productivity and fishing intensity) could also potentially influence the structure of coral reef fish communities and their response to heat stress. Also, the grouping of continuous traits into static, categorical groups reduced the value of those indices that reflect the continuum and diversity of trait representation among species, potentially inhibiting the ability of our analysis to provide insights into response diversity along dynamic trait gradients.

Here we modelled coral reef fish species responses to a severe marine heatwave at a continental scale. By incorporating species functional traits, we were also able to identify those traits that characterize the ‘winners’ (e.g. small-bodied herbivores) and ‘losers’ (e.g. highly coral-associated species) among fish species to different facets of heat stress. Finally, we identified responses for four functionally distinct communities in terms of change in proportional trait richness, which indicated that coral reef fish communities at lower latitudes are likely more vulnerable to future marine heatwaves and could benefit from strategic conservation efforts. For example, larger-bodied species may need to be better protected through more conservative fisheries management or expanded areas of no-take protection. This knowledge has broadened our understanding of functional responses to thermal disturbances beyond local scales that are most commonly the focus of current conservation efforts. Future research should consider additional functional traits to account for other critical ecological functions fishes provide within coral reef communities.

## Supplementary Material



## Data Availability

All data and code required to replicate the analysis and outputs of this study are available at Mendeley Data [30]. Supplementary material is available online [60].
