# Supplementary material for: Cumulative thermal stress predicts functional change in coral reef fish communities
Source: Proc Biol Sci. 2026 Oct 28;293(2081):20261955. doi: 10.1098/rspb.2026.1955 (PMC13725540; doi:10.1098/rspb.2026.1955)
Supplement: Supplementary file 1 [file rspb.2026.1955.zip › rspb.2026.1955/rspb20261955_si_001.pdf]

OFFICIAL

Supplementary Material

Cumulative thermal stress predicts functional change in coral reef fish communities

Markey, A.C., Fordham, D.A., Stuart-Smith, R.D., Edgar, G.J., Brown, C.J. Mellin, C.

Proceedings of the Royal Society B

DOI: 10.1098/rspb.2026.1955

OFFICIAL

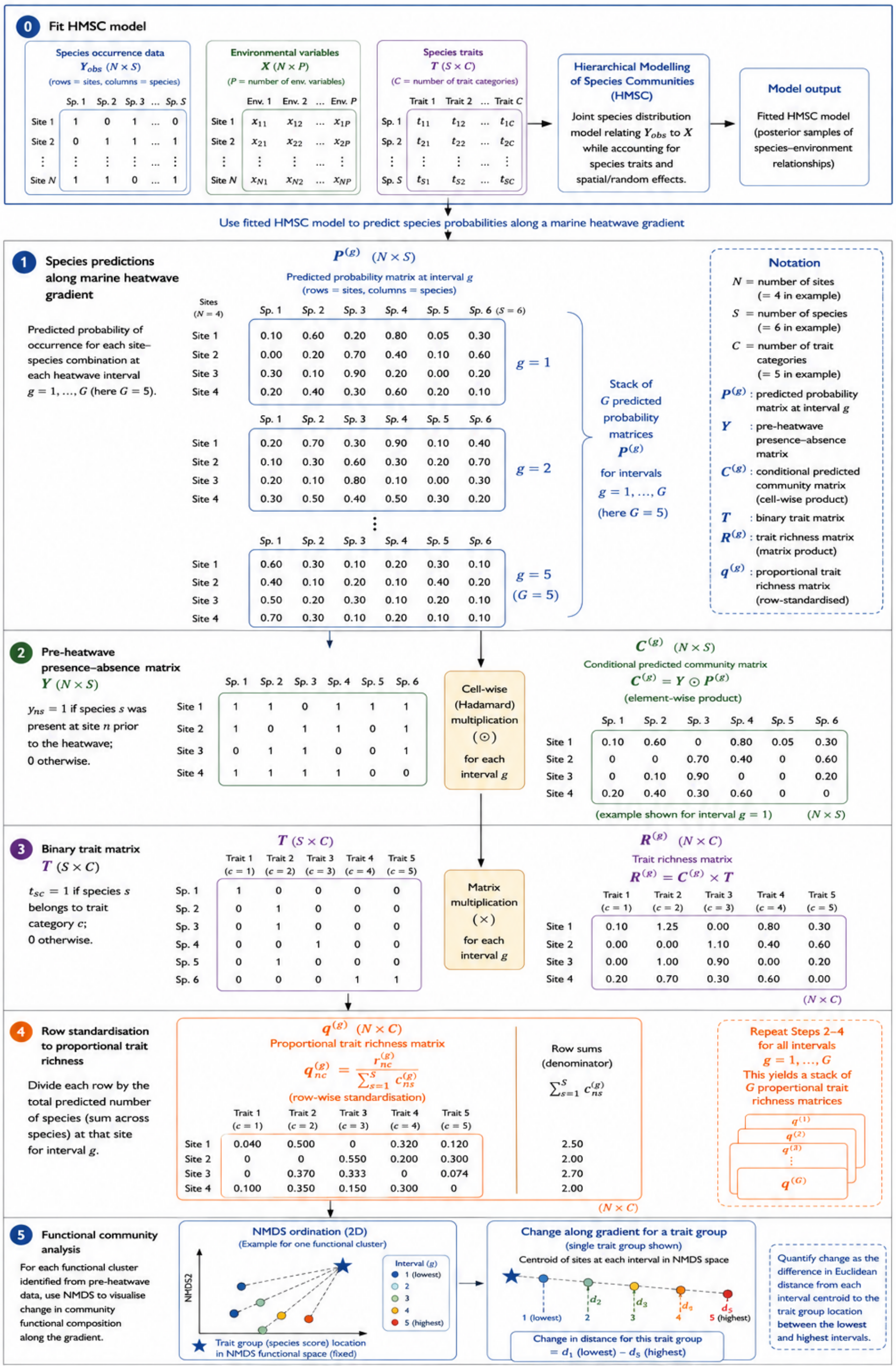

Figure S1: Flow diagram of the functional community analysis using HMSC generated species predictions and functionally distinct community clusters.

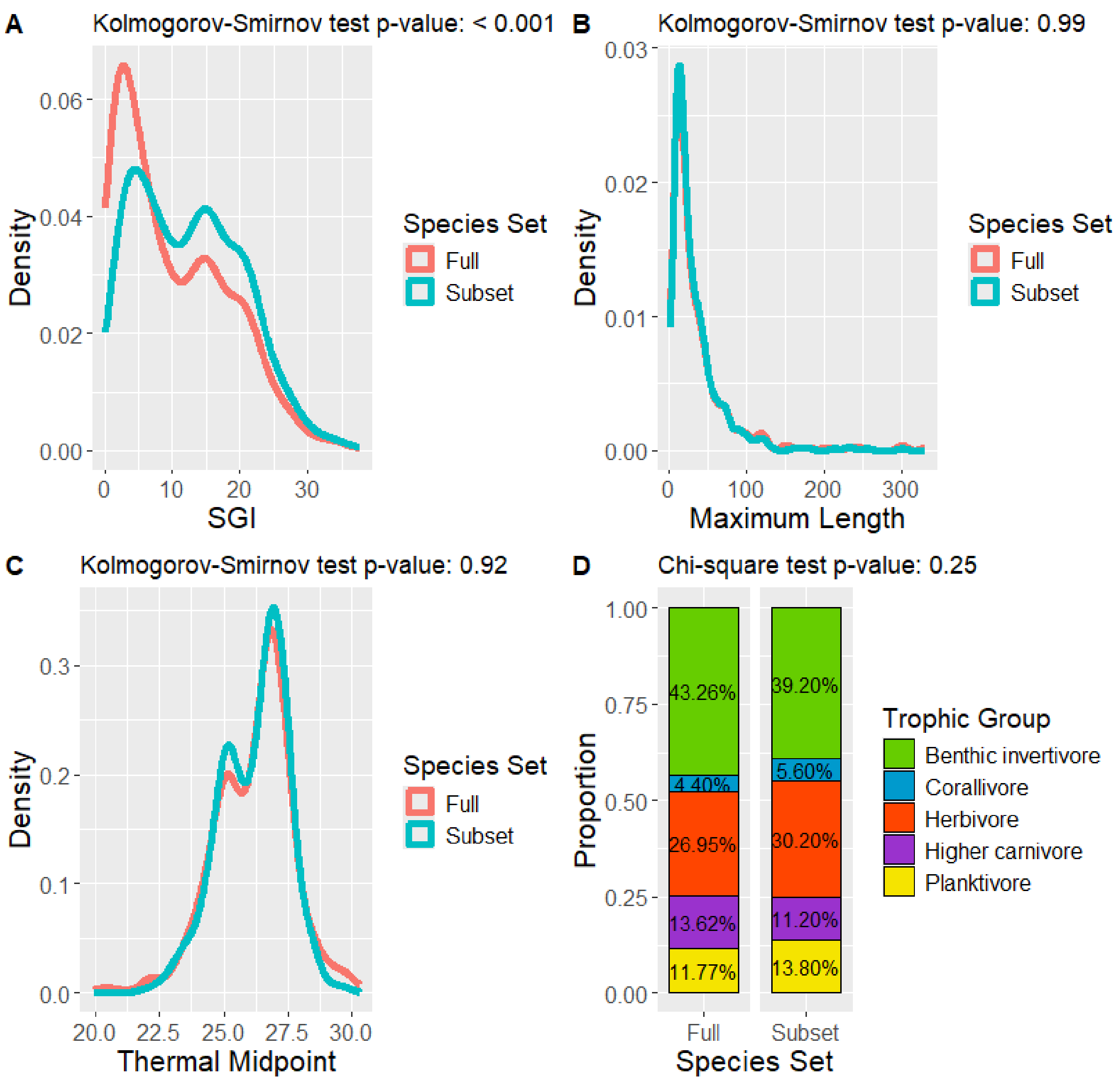

Figure S2: Density (numeric traits) and mosaic (trophic group) plots of species trait distributions for the full and subset of species for A) species generalisation index (SGI), B) maximum length, C) thermal midpoint and D) trophic group. Titles contain p-values for each traits respective distribution test.

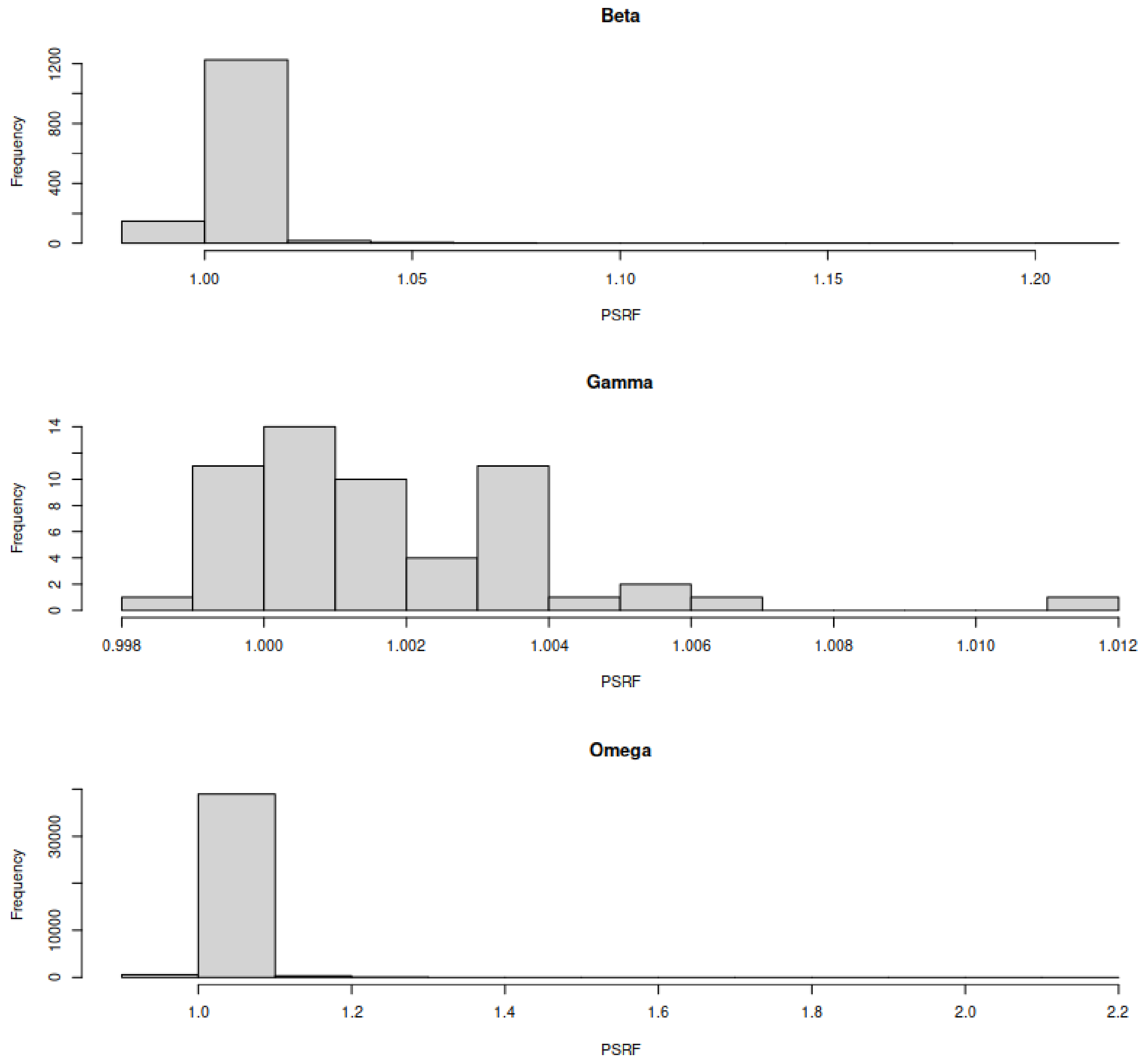

Figure S3: Histograms of post scale reduction factors for β, Γ and Ω parameters.

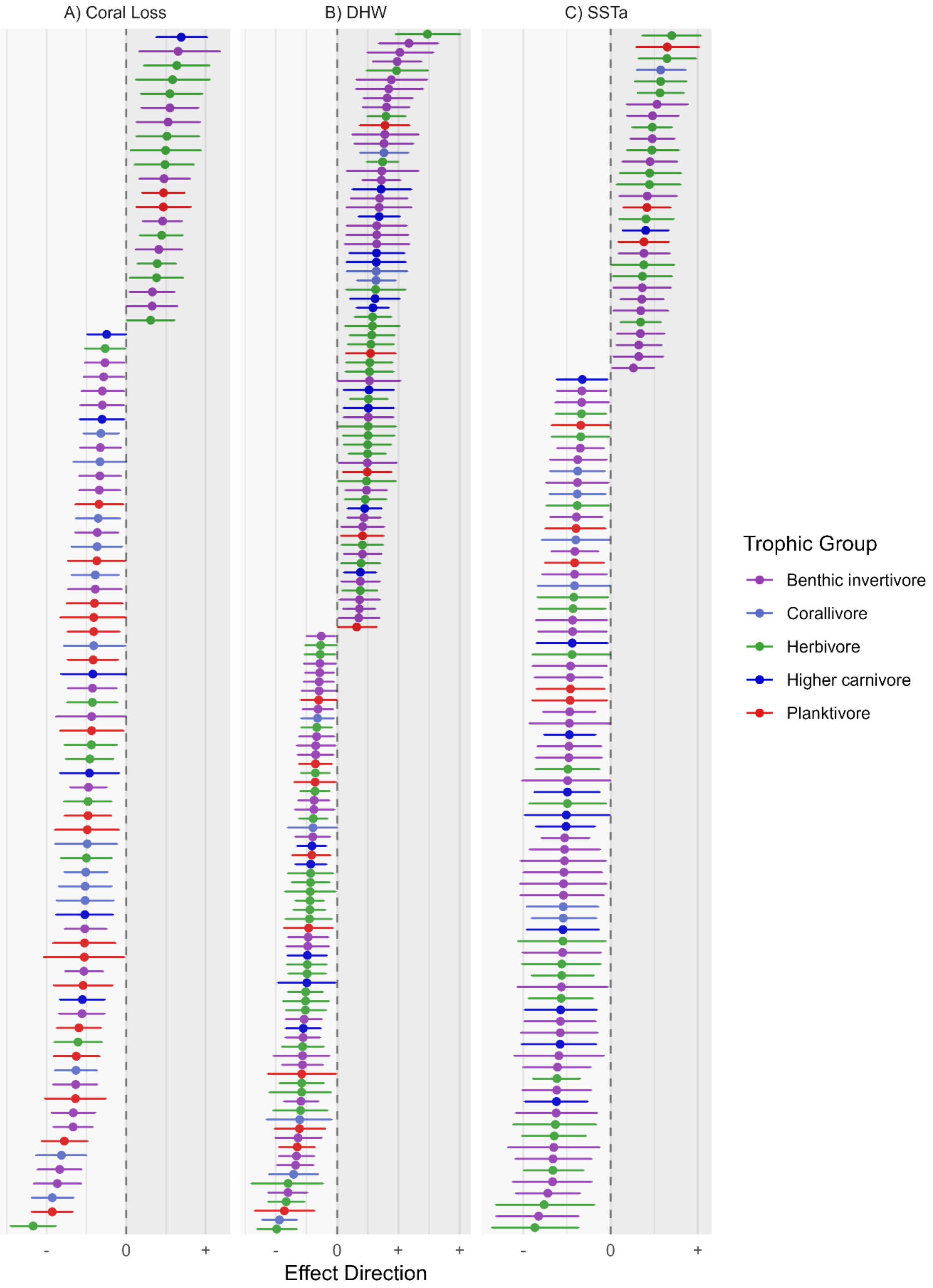

Figure S4: Forest plots of β (species-environment) effect sizes for (A) coral loss , (B) degree heating weeks (DHW) and (C) sea surface temperature anomaly (SSTa). Error bars represent 95% credible intervals. Points are coloured by trophic group. Only species with high evidence for an effect are displayed (i.e. 95% credible intervals do not overlap 0).

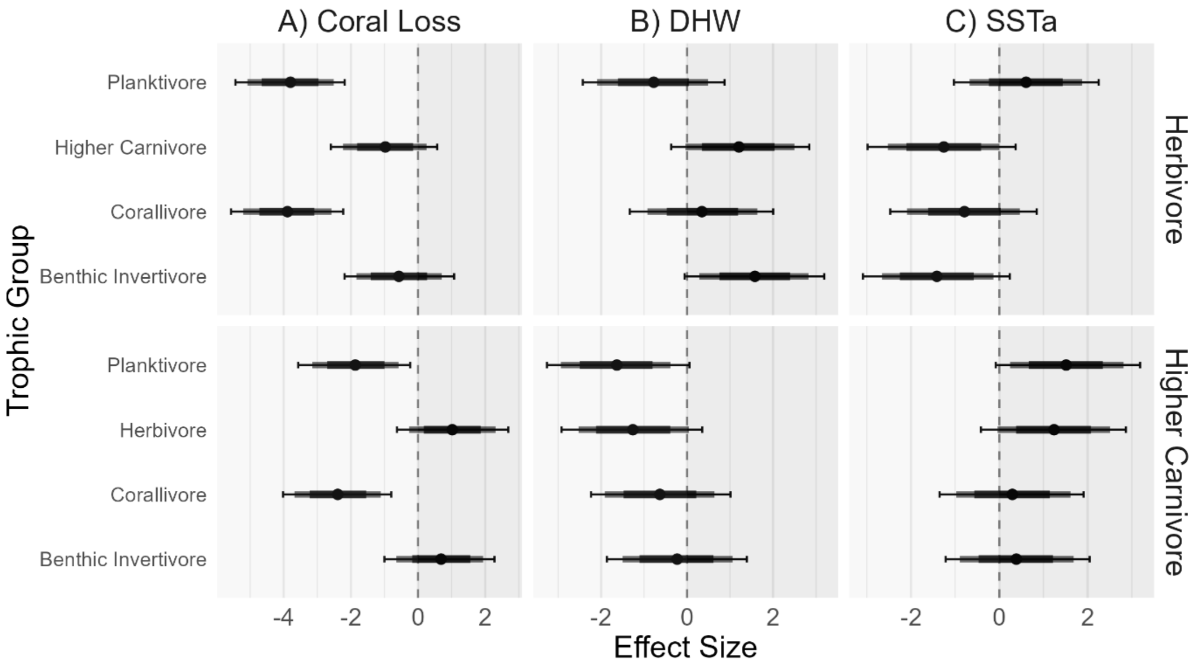

Figure S5: Forest plots of trophic group Γ (trait-environment) effect sizes for (A) coral loss, (B) degree heating weeks and (C) sea surface temperature anomaly, split by reference groups of herbivore (top) and higher carnivore (bottom). Posterior distributions have been scaled by dividing by their standard deviations. Displayed intervals in order of increasing thickness are 95, 90 & 80% credible intervals.

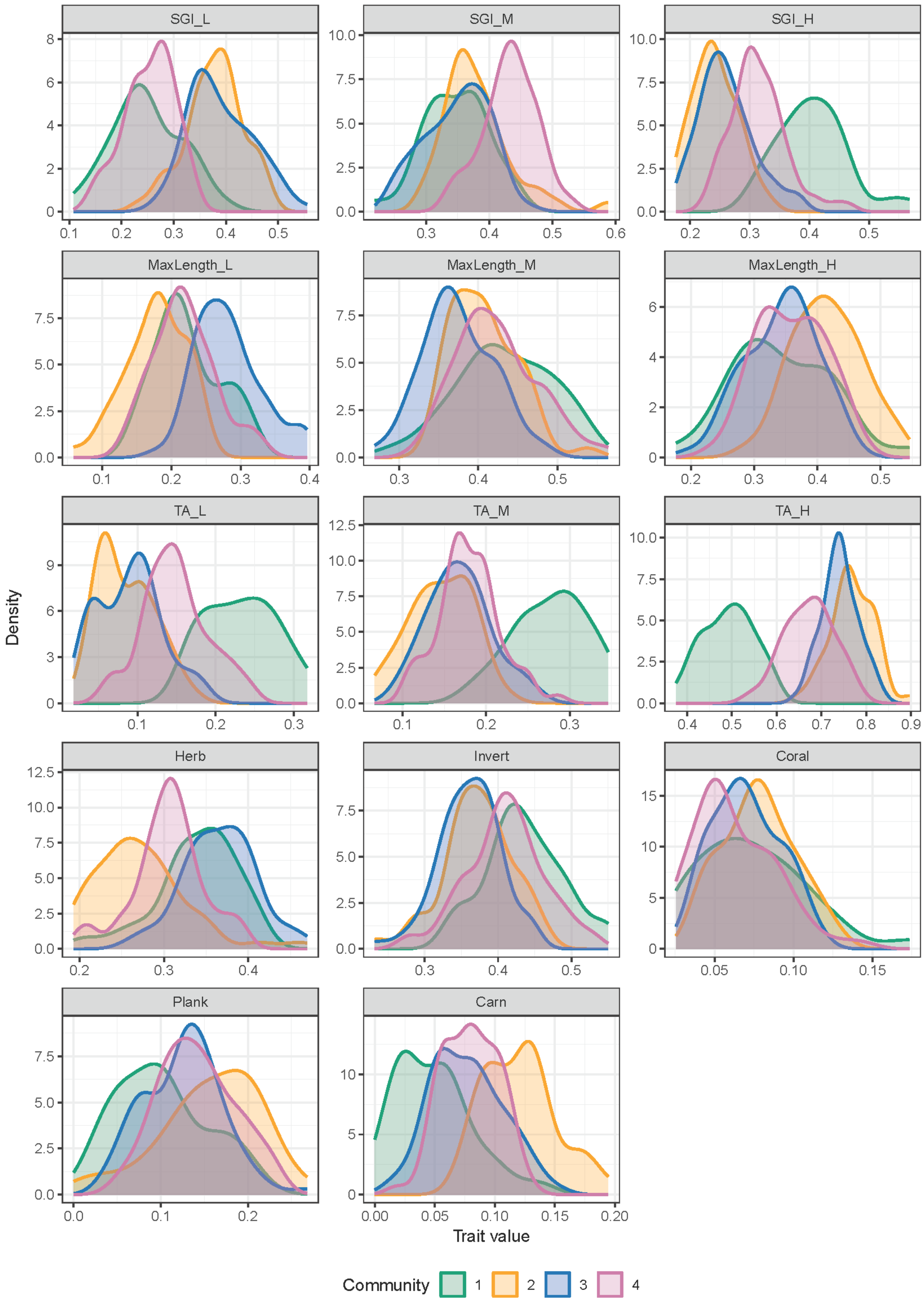

Figure S6: Density plots of proportional trait richness for each trait group used in our functional analysis. Density lines and hulls are coloured by community cluster.

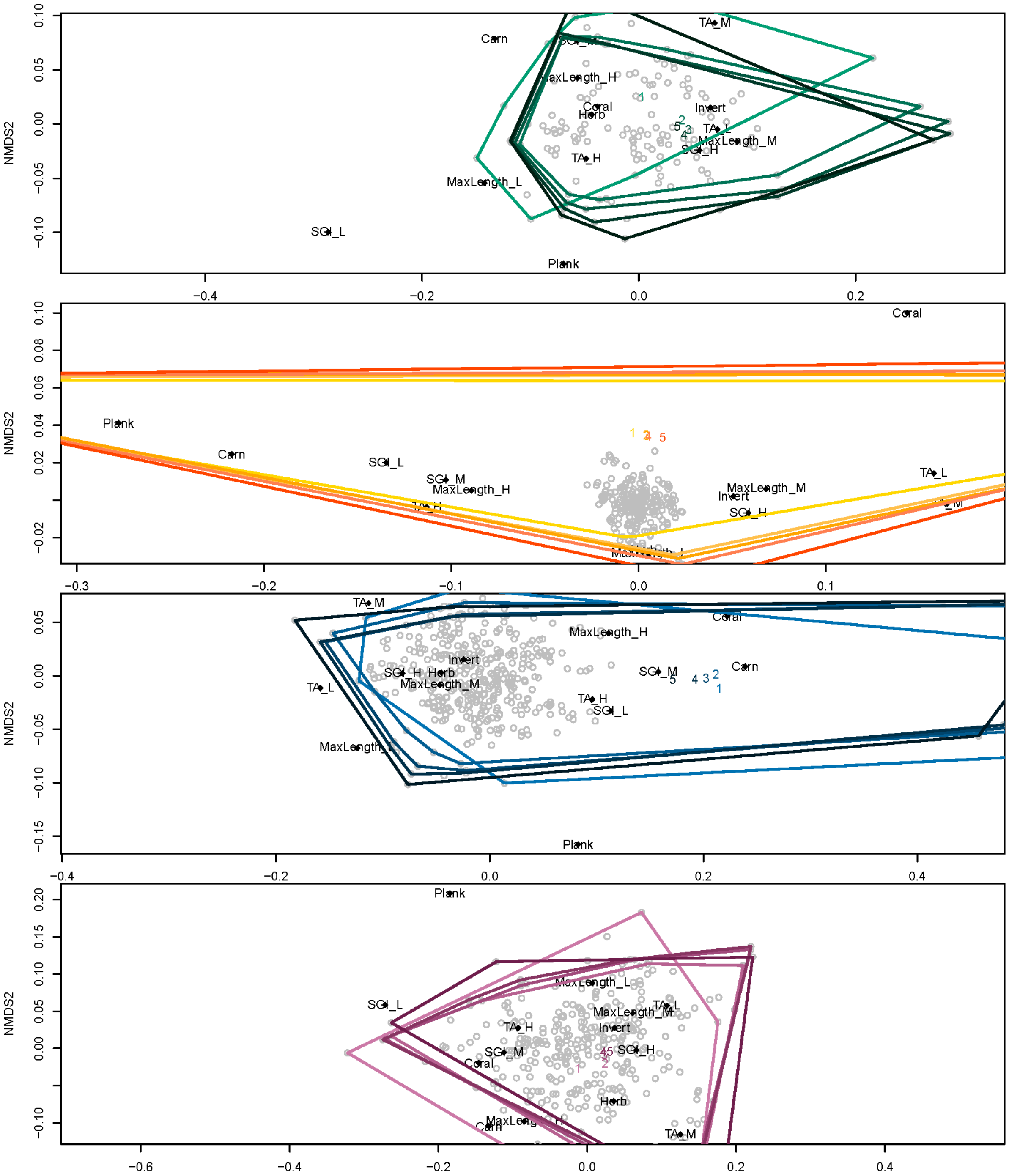

Figure S7: NMDS bi-plots of communities trait composition predictions along a gradient of marine heatwave intensity for communities A) Southern, B) Outer, C) Northern and D) Coral Sea. Hulls represent each interval along the marine heatwave gradient and colours get darker as the heatwave intensity increases. Trait groups are labelled with text.

Table S1: All 500 modelled species and their scaled mean β (species-environment) response to three heatwave related variables: degree heating weeks (DHW), sea surface temperature anomaly (SSTa) and live coral cover loss, as well as their predicted change in probability of occurrence between a no heatwave state and median heatwave state (MHW gradient). Values with ***, **, and * indicate 95%, 90%, and 80% credible intervals not overlapping zero, respectively.

Species	DHW	SSTA	Coral Loss	MHW Gradient
Abudefduf bengalensis	-0.037*	0.21	-0.0073**	-0.085
Abudefduf sexfasciatus	-0.061***	0.65***	-0.003	0.033**
Abudefduf vaigiensis	0.0043	-0.29**	-0.00057	-0.1*
Abudefduf whitleyi	-0.052**	0.23	-0.00051	-0.0051
Acanthochromis polyacanthus	0.078***	-0.46***	-0.0041*	-0.11**
Acanthurus albipectoralis	-0.043**	0.036	0.004	-0.0044
Acanthurus auranticavus	-0.026*	-0.13	-0.00045	-0.021*
Acanthurus blochii	0.0072	-0.27**	-0.0016	-0.2**
Acanthurus dussumieri	-0.056***	0.47***	0.0044*	0.19***
Acanthurus grammoptilus	0.08***	-0.33***	-0.0012	-0.082
Acanthurus leucocheilus	0.097***	-0.63***	0.0063*	-0.035
Acanthurus lineatus	0.016*	0.012	0.0038*	0.034*
Acanthurus nigricans	0.058***	-0.052	-0.0096***	-0.092*
Acanthurus nigricauda	0.00025	0.24**	0.0033*	0.073***
Acanthurus nigrofuscus	-0.035***	0.13	0.0078***	0.12**
Acanthurus nigroris	-0.058***	0.25*	-0.0029	-0.0036
Acanthurus olivaceus	0.039***	-0.2*	0.00049	-0.022
Acanthurus pyroferus	0.021*	-0.13	-0.0036*	-0.031*
Acanthurus thompsoni	-0.005	0.33**	-0.008***	-0.0099
Acanthurus triostegus	-0.027*	0.27*	0.00016	0.0042
Acanthurus xanthopterus	0.051***	-0.42**	-0.0031	-0.17**
Aethaloperca rogaa	0.062***	-0.57***	-0.0017	-0.2**
Amblyeleotris guttata	0.049**	-0.34*	0.0039	-0.011
Amblyeleotris steinitzi	-0.011	-0.11	0.0022	-0.038
Amblyeleotris wheeleri	0.082***	-0.43***	0.0046*	-0.023
Amblyglyphidodon aureus	0.042**	-0.32*	0.0099***	0.017
Amblyglyphidodon curacao	-0.045***	0.15	-0.0055**	-0.074*
Amblyglyphidodon leucogaster	0.043**	-0.29*	0.0035	-0.0082

Species	DHW	SSTA	Coral Loss	MHW Gradient
Amblyglyphidodon ternatensis	0.044*	-0.47**	0.0026	-0.039
Amblygobius decussatus	0.016	0.078	-0.0012	-0.0018
Amblygobius nocturnus	0.024	-0.19	0.004	-0.011
Amblygobius phalaena	-0.03**	-0.15	0.0053**	-0.033
Amphiprion akindynos	-0.018	-0.14	-0.00028	-0.072
Amphiprion chrysopterus	-0.017	-0.14	0.003	-0.02
Amphiprion clarkii	-0.0016	-0.39***	-0.00059	-0.24***
Amphiprion melanopus	0.02	-0.12	-0.0068**	-0.032*
Amphiprion perideraion	-0.019	-0.18	0.0026	-0.081
Anampses caeruleopunctatus	-0.021*	-0.2*	-0.0034	-0.072**
Anampses femininus	-0.045**	0.043	-0.001	-0.0011
Anampses geographicus	-0.0048	0.29**	0.0042*	0.06***
Anampses meleagrides	0.0088	0.013	-0.0023	-0.02
Anampses neoguinaicus	-0.064***	0.21*	-0.005**	-0.032
Anampses twistii	-0.033***	0.36***	0.0045*	0.019***
Anyperodon leucogrammicus	0.0054	0.017	-0.0092***	-0.043*
Aphareus furca	-0.029**	0.097	-0.0039*	-0.048
Apogon doederleini	-0.019	-0.2	0.0047*	-0.045
Apolemichthys trimaculatus	0.065***	-0.25	0.0036	0.0022
Aprion virescens	0.006	-0.2*	-0.006**	-0.079**
Arothron hispidus	0.0083	-0.043	-0.0019	-0.042
Arothron nigropunctatus	0.011	-0.14	-0.0029*	-0.15*
Arothron stellatus	-0.036*	0.073	0.0036	-0.0063
Assessor flavissimus	-0.0017	-0.064	0.0023	-0.01
Assessor macneilli	0.032*	-0.075	0.0013	0.02
Aulostomus chinensis	-0.048***	0.32**	-0.011***	-0.054*
Balistapus undulatus	-0.012	0.38***	0.0033*	0.17***
Balistoides conspicillum	0.02*	-0.3**	-0.00024	-0.062*
Balistoides viridescens	0.031**	0.05	-0.0011	0.0007
Belonoperca chabanaudi	0.018	-0.14	-0.0019	-0.061
Bodianus axillaris	-0.021**	0.26***	-0.0033*	0.041
Bodianus diana	0.015	-0.39**	-0.0042	-0.11**
Bodianus dictynna	0.047**	-0.026	-0.0026	-0.0061
Bodianus loxozonus	0.0011	-0.25*	0.0014	-0.026

Species	DHW	SSTA	Coral Loss	MHW Gradient
Bodianus mesothorax	0.039**	-0.36**	0.00085	-0.067
Bolbometopon muricatum	0.0068	-0.056	-0.004	-0.06
Bryaninops natans	0.0098	-0.27*	0.0014	-0.03
Caesio caerulaurea	-0.023*	-0.097	-0.0082***	-0.13***
Caesio cuning	0.049***	-0.34***	-0.0013	-0.15*
Caesio lunaris	0.002	0.13	-0.0046*	-0.01
Caesio teres	0.013	0.19*	-0.005*	-0.0012
Calotomus carolinus	0.018	-0.41**	0.0031	-0.027
Cantherhines dumerilii	0.017	-0.42**	-0.0065*	-0.13***
Cantherhines pardalis	0.0037	-0.069	-0.0034*	-0.074
Canthigaster bennetti	0.032*	-0.46**	0.0086**	-0.0086
Canthigaster janthinoptera	0.039*	-0.54***	0.0033	-0.019*
Canthigaster papua	0.053***	-0.23*	-0.0046*	-0.11*
Canthigaster valentini	0.02*	-0.11	0.0021	-0.0019
Carangoides fulvoguttatus	0.019	-0.38**	0.0043	-0.087
Carangoides orthogrammus	0.038**	-0.44***	0.00077	-0.12*
Carangoides plagiotaenia	-0.0069	-0.2	-0.0022	-0.12*
Caranx ignobilis	0.04**	-0.54***	0.0029	-0.081*
Caranx lugubris	0.031*	-0.35*	0.00027	-0.056
Caranx melampygus	0.059***	-0.47***	-0.0029*	-0.25***
Carcharhinus amblyrhynchos	-0.012	0.13	0.0024	0.025
Centropyge bicolor	0.057***	-0.058	0.0018	0.059
Centropyge bispinosa	-0.03**	-0.047	-0.0085***	-0.092***
Centropyge eibli	-0.037**	0.33*	0.0048*	0.064**
Centropyge flavissima	-0.0045	-0.18	-0.001	-0.021
Centropyge heraldi	-0.031*	0.14	0.0012	-0.00074
Centropyge tibicen	-0.0055	-0.43***	0.00015	-0.033***
Centropyge vrolikii	0.074***	-0.61***	0.0021	-0.17**
Cephalopholis argus	-0.018*	0.028	-0.0023	-0.06
Cephalopholis boenak	0.038*	-0.32*	-0.00034	-0.054
Cephalopholis cyanostigma	0.052***	-0.51***	-0.0033	-0.27***
Cephalopholis leopardus	0.051***	-0.29*	-0.0047*	-0.087*
Cephalopholis miniata	0.015	-0.37**	-0.0015	-0.11*
Cephalopholis spiloparaea	0.017	-0.45**	-0.0035	-0.1**

Species	DHW	SSTA	Coral Loss	MHW Gradient
Cephalopholis urodeta	0.069***	-0.62***	0.0014	-0.12**
Cetoscarus bicolor	-0.037**	0.2	0.0034	0.016
Chaetodon adiergastos	0.076***	-0.41***	-0.004*	-0.12*
Chaetodon aureofasciatus	0.04**	-0.54***	-0.0059**	-0.29***
Chaetodon auriga	-0.019**	-0.15*	-0.0064***	-0.3***
Chaetodon baronessa	0.013	-0.2*	-0.016***	-0.31***
Chaetodon bennetti	-0.016	-0.28*	-0.00065	-0.12**
Chaetodon citrinellus	0.0081	-0.043	0.0012	0.0091
Chaetodon ephippium	0.012	-0.063	-0.005**	-0.076*
Chaetodon flavirostris	-0.039***	-0.23*	-0.0015	-0.16***
Chaetodon kleinii	0.014	-0.25*	-0.0066***	-0.17***
Chaetodon lineolatus	0.013	-0.2*	-0.0054**	-0.2**
Chaetodon lunula	0.0038	0.21*	-0.0035*	0.021
Chaetodon lunulatus	-0.026***	0.22**	-0.011***	-0.15**
Chaetodon melannotus	-0.016*	-0.15*	-0.006***	-0.12***
Chaetodon mertensii	-0.026*	0.092	0.00018	-0.0043
Chaetodon meyeri	0.026*	-0.22	-0.0032	-0.057
Chaetodon ornatissimus	-0.01	0.27*	-0.01***	-0.056
Chaetodon pelewensis	-0.071***	0.34**	-0.0073***	-0.02
Chaetodon plebeius	-0.016	-0.072	-0.007***	-0.19***
Chaetodon punctatofasciatus	-0.056***	0.31*	-0.0059*	-0.036
Chaetodon rafflesii	0.064***	-0.19	-0.00066	-0.015
Chaetodon rainfordi	-0.032*	-0.02	-0.0098***	-0.19***
Chaetodon reticulatus	0.027*	-0.32*	-0.0029	-0.048*
Chaetodon semeion	-0.0079	0.066	-0.0062**	-0.039
Chaetodon speculum	-0.021*	0.13	-0.0056**	-0.084
Chaetodon trifascialis	-0.032***	0.23**	-0.013***	-0.15***
Chaetodon ulietensis	-0.028***	0.041	-0.0073***	-0.17**
Chaetodon unimaculatus	-0.038***	0.1	-0.011***	-0.16***
Chaetodon vagabundus	0.063***	-0.38***	-0.0078***	-0.21***
Chaetodontoplus duboulayi	0.043*	-0.53***	0.0069*	-0.04
Chaetodontoplus meredithi	-0.0016	-0.46**	0.0013	-0.12**
Chaetodontoplus mesoleucus	0.073***	-0.59***	0.0016	-0.049*
Cheilinus chlorourus	-0.011	0.12	0.0021	0.053*

Species	DHW	SSTA	Coral Loss	MHW Gradient
Cheilinus fasciatus	0.038***	-0.39***	0.0025	-0.098
Cheilinus oxycephalus	0.051***	-0.48***	0.0013	-0.063*
Cheilinus trilobatus	-0.035***	0.32***	-0.0012	0.018
Cheilinus undulatus	-0.027**	0.14	-0.0037*	-0.046
Cheilodipterus artus	0.039**	-0.32**	0.0003	-0.05
Cheilodipterus isostigmus	0.000032	-0.0099	-0.004	-0.029
Cheilodipterus macrodon	-0.036***	0.27**	-0.004*	-0.013
Cheilodipterus quinquelineatus	-0.009	0.057	-0.0013	-0.017
Chelmon marginalis	0.032*	-0.54***	-0.00052	-0.22***
Chelmon rostratus	0.018	-0.22	0.0015	-0.034
Chlorurus bleekeri	0.014	-0.18*	0.00083	-0.037
Chlorurus microrhinos	-0.044***	0.48***	0.00091	0.2***
Chlorurus sordidus	-0.044***	0.57***	-0.0042*	0.17**
Choerodon cyanodus	0.012	-0.15	0.0052*	0.011
Choerodon fasciatus	0.013	-0.21*	-0.0028	-0.13*
Choerodon graphicus	-0.0063	-0.23	-0.0032	-0.061*
Choerodon schoenleinii	0.026*	-0.46***	-0.00073	-0.26**
Chromileptes altivelis	0.035*	-0.58***	-0.00029	-0.2***
Chromis agilis	-0.024*	-0.093	-0.0087***	-0.039***
Chromis alpha	0.032*	0.038	-0.0027	-0.0044
Chromis amboinensis	0.0017	-0.07	-0.0061**	-0.077*
Chromis atripectoralis	-0.065***	0.42***	-0.019***	-0.14***
Chromis atripes	-0.01	0.27**	-0.0096***	-0.024
Chromis chrysura	-0.086***	0.098	-0.003	-0.017*
Chromis flavomaculata	-0.058***	-0.0069	-0.0092**	-0.0052***
Chromis iomelas	-0.027*	-0.054	-0.011***	-0.074***
Chromis lepidolepis	-0.0012	-0.27**	-0.0035*	-0.16***
Chromis margaritifer	-0.026**	0.14	-0.0069***	-0.076*
Chromis nitida	-0.031	-0.39**	-0.0038	-0.19***
Chromis retrofasciata	-0.00064	0.022	-0.013***	-0.097**
Chromis ternatensis	-0.011	0.2*	-0.016***	-0.14***
Chromis vanderbilti	-0.046***	0.012	-0.0032	-0.026*
Chromis viridis	-0.041***	0.38***	-0.012***	-0.075*
Chromis weberi	0.013	-0.22*	-0.0042*	-0.17**

Species	DHW	SSTA	Coral Loss	MHW Gradient
Chromis xanthochira	-0.02	0.12	-0.0036	-0.014
Chromis xanthura	0.018*	-0.23*	-0.0025	-0.062*
Chrysiptera biocellata	0.063***	-0.2	0.0032	0.002
Chrysiptera brownriggii	0.04**	-0.28*	0.009**	-0.0012
Chrysiptera cyanea	-0.035*	0.17	0.0014	0.00075
Chrysiptera flavipinnis	0.01	-0.49***	0.012***	-0.014
Chrysiptera hemicyanea	-0.0018	-0.097	-0.0032	-0.041
Chrysiptera rex	0.15***	-0.87***	0.002	-0.046*
Chrysiptera rollandi	-0.016	-0.05	0.0017	-0.012
Chrysiptera talboti	-0.017	0.013	-0.001	-0.018
Chrysiptera taupou	-0.014	-0.12	0.0029	-0.011
Cirrhilabrus exquisitus	0.081***	-0.48***	-0.000011	-0.091
Cirrhilabrus laboutei	-0.0012	-0.22	0.0045	-0.011
Cirrhilabrus punctatus	-0.03*	-0.15	-0.0016	-0.066*
Cirrhilabrus randalli	0.051**	-0.28*	-0.0024	-0.05
Cirrhitichthys falco	-0.017	-0.081	0.0039	-0.0073
Cirrhitichthys oxycephalus	0.049***	-0.47**	0.013***	0.018
Cirripectes castaneus	0.0084	-0.12	-0.0014	-0.051
Cirripectes filamentosus	0.0058	0.36**	-0.0022	0.033*
Cirripectes stigmaticus	-0.038**	-0.24*	0.011***	-0.0041
Coris aygula	-0.08***	0.45***	-0.0041*	-0.003
Coris batuensis	0.019	-0.3**	0.0061**	-0.0083
Coris dorsomacula	0.0061	-0.4**	0.00051	-0.041*
Coris gaimard	-0.0013	-0.075	0.0016	-0.011
Crossosalarias macrospilus	-0.014	-0.29*	0.0089**	-0.013
Cryptocentrus strigilliceps	0.069***	-0.3*	0.0058*	0.014
Ctenochaetus binotatus	-0.0075	-0.099	-0.00071	-0.087
Ctenochaetus cyanocheilus	0.041***	-0.38***	0.004*	-0.015
Ctenochaetus striatus	-0.017*	0.086	0.0013	0.057
Ctenogobiops mitodes	0.0059	-0.58***	0.0043	-0.14**
Ctenogobiops pomastictus	0.035**	-0.1	-0.0032	-0.045
Cypho purpurascens	0.0012	-0.32**	0.0049*	-0.051
Dascyllus aruanus	-0.052***	0.41***	-0.0091***	-0.026
Dascyllus reticulatus	-0.035***	0.17*	0.00023	0.012

Species	DHW	SSTA	Coral Loss	MHW Gradient
Dascyllus trimaculatus	-0.03***	-0.068	-0.00055	-0.061
Diagramma labiosum	0.021	-0.22*	0.001	-0.067
Diploprion bifasciatum	0.0067	-0.53***	0.00045	-0.24***
Dischistodus melanotus	-0.008	-0.023	-0.0025	-0.029
Dischistodus perspicillatus	0.024*	-0.04	0.0087**	0.053**
Dischistodus prosopotaenia	0.053***	-0.3**	0.0097***	0.065*
Dischistodus pseudochrysopoecilus	0.0039	-0.21*	0.007**	0.00028
Echeneis naucrates	-0.0046	-0.27*	-0.0015	-0.092*
Ecsenius aequalis	0.029	-0.31*	0.0049	-0.0068
Ecsenius alleni	0.041**	-0.13	0.0015	-0.0009
Ecsenius bicolor	0.019*	-0.18*	0.0038*	0.017
Ecsenius lividanalis	0.055**	-0.46**	-0.00046	-0.016*
Ecsenius schroederi	0.038*	0.01	0.00027	-0.00089
Ecsenius stictus	0.028*	-0.49***	-0.0028	-0.23***
Ecsenius yaeyamaensis	0.019	0.017	0.00065	-0.0012
Elagatis bipinnulata	0.064***	-0.51***	-0.0031	-0.14*
Epibulus insidiator	-0.029***	0.32***	-0.0057***	0.0052
Epinephelus fasciatus	-0.048***	-0.072	0.00026	-0.13*
Epinephelus malabaricus	-0.029	-0.1	0.0043	-0.026
Epinephelus merra	0.014	-0.14	0.014***	0.091***
Epinephelus polyphekadion	-0.017	-0.038	0.0018	-0.022
Epinephelus quoyanus	0.017	-0.38**	0.005*	-0.042
Epinephelus spilotoceps	0.0086	-0.047	0.000062	-0.016
Epinephelus tauvina	-0.0096	-0.11	0.0038	-0.02
Eviota atriventris	0.084***	-0.32*	0.006*	0.013
Eviota guttata	-0.054***	-0.33***	-0.0013	-0.27***
Eviota prasites	0.02	-0.39**	0.0017	-0.11*
Eviota sebreei	0.018	-0.33*	-0.0038	-0.14**
Exallias brevis	-0.061***	-0.22	-0.0036	-0.097***
Exyrias belissimus	0.089***	-0.56***	0.0018	-0.03
Fistularia commersonii	-0.013	0.27*	-0.0037	-0.0029
Forcipiger flavissimus	-0.018*	0.12	0.0048**	0.035*
Forcipiger longirostris	-0.0022	-0.037	-0.00047	-0.027
Fusigobius duospilus	-0.0013	-0.33*	-0.0019	-0.059**

Species	DHW	SSTA	Coral Loss	MHW Gradient
Fusigobius neophytus	-0.036*	-0.082	-0.00061	-0.054
Fusigobius signipinnis	0.038**	-0.31*	-0.0041*	-0.09*
Gnathodentex aureolineatus	0.042***	-0.19*	-0.0049*	-0.052*
Gnatholepis anjerensis	0.013	-0.44***	0.0019	-0.077*
Gnatholepis cauerensis	-0.013	-0.022	0.005*	0.011
Gobiodon spilophthalmus	0.041*	-0.3*	0.00029	-0.031
Gomphosus varius	-0.0086	0.36***	-0.017***	-0.19**
Grammatorcynus bilineatus	0.072***	-0.4**	-0.0032	-0.12*
Gymnocranius euanus	-0.0041	-0.42**	-0.0028	-0.11***
Gymnothorax javanicus	-0.024*	-0.19	0.0025	-0.08
Halichoeres biocellatus	-0.0045	0.024	0.0015	0.024
Halichoeres chloropterus	0.038*	-0.57***	0.0055*	-0.072*
Halichoeres chrysus	0.065***	-0.54***	0.0066*	-0.032
Halichoeres hortulanus	-0.04***	0.19*	0.0042**	0.093*
Halichoeres margaritaceus	0.011	-0.19	0.0048*	-0.01
Halichoeres marginatus	-0.029***	0.0058	-0.00036	-0.056
Halichoeres melanochir	0.07***	-0.2	0.0058*	0.02
Halichoeres melanurus	0.098***	-0.72***	-0.005*	-0.21***
Halichoeres nebulosus	0.014	-0.38***	0.0038*	-0.095
Halichoeres nigrescens	0.038*	-0.66***	0.004	-0.17**
Halichoeres prosopeion	0.024*	-0.27*	0.0014	-0.034
Halichoeres trimaculatus	-0.022*	-0.11	0.0054**	-0.0068
Hemiglyphidodon plagiometopon	0.0047	-0.0000068	-0.01***	-0.093**
Hemigymnus fasciatus	-0.028***	0.2**	-0.0095***	-0.17**
Hemigymnus melapterus	-0.055***	0.34***	-0.0066***	-0.051
Hemitaurichthys polylepis	0.026*	-0.064	-0.0062*	-0.039
Heniochus acuminatus	-0.026*	0.21*	-0.0074***	-0.057
Heniochus chrysostomus	0.0019	0.0011	-0.0067***	-0.11*
Heniochus monoceros	0.0029	-0.093	-0.0044*	-0.086*
Heniochus singularius	0.0037	-0.19	-0.0011	-0.029
Heniochus varius	-0.035***	0.35***	-0.013***	-0.12*
Hipposcarus longiceps	0.02**	0.05	0.0028*	0.052*
Hologymnosus annulatus	-0.049***	0.24*	-0.0044*	-0.023
Hologymnosus doliatus	0.065***	-0.62***	-0.00027	-0.11**

Species	DHW	SSTA	Coral Loss	MHW Gradient
Istigobius decoratus	0.022*	-0.22*	0.0056**	-0.00035
Istigobius rigilius	0.05***	-0.57***	-0.0039*	-0.26***
Koumansetta rainfordi	-0.051***	0.23*	0.001	0.02
Kyphosus cinerascens	-0.000089	-0.26*	-0.0037	-0.072**
Kyphosus vaigiensis	0.048***	-0.56***	-0.00075	-0.12**
Labracinus cyclophthalmus	0.078***	-0.54***	0.0071*	-0.017
Labrichthys unilineatus	-0.094***	0.57***	-0.019***	-0.17***
Labroides bicolor	-0.038***	0.085	-0.0065***	-0.13**
Labroides dimidiatus	-0.012	-0.11	-0.0049**	-0.065***
Labroides pectoralis	0.004	0.094	-0.0044*	-0.024
Labropsis australis	-0.026*	-0.4***	-0.01***	-0.29***
Labropsis xanthonota	-0.014	-0.017	-0.0082***	-0.11*
Lepidozygus tapeinosoma	0.031	-0.26	-0.0068*	-0.064*
Lethrinus atkinsoni	0.017	-0.21	-0.000049	-0.051
Lethrinus erythropterus	0.018	-0.083	0.0029	0.00091
Lethrinus nebulosus	-0.02	-0.18	0.0088**	-0.0035
Lethrinus obsoletus	0.012	-0.39**	0.0029	-0.058*
Lethrinus olivaceus	0.012	-0.17	0.0021	-0.041
Lutjanus bohar	-0.041***	0.065	-0.0049***	-0.17**
Lutjanus carponotatus	0.0024	-0.49***	-0.01***	-0.43***
Lutjanus decussatus	0.064***	-0.3*	-0.0084***	-0.11*
Lutjanus fulviflamma	-0.0062	0.016	0.001	-0.0058
Lutjanus fulvus	0.045**	-0.3*	-0.0066*	-0.088*
Lutjanus gibbus	0.045***	-0.32***	-0.00086	-0.1*
Lutjanus kasmira	0.013	-0.052	-0.0078***	-0.095*
Lutjanus monostigma	0.022	-0.11	0.002	-0.014
Lutjanus quinquelineatus	-0.027	0.1	-0.0021	-0.018
Lutjanus rivulatus	-0.025*	0.37**	0.0052*	0.013**
Lutjanus russellii	-0.0016	-0.15	0.0034	-0.018
Macolor macularis	0.027*	-0.41***	0.0001	-0.089*
Macolor niger	-0.022**	0.18*	-0.00059	0.0053
Macropharyngodon choati	-0.0038	-0.32*	0.0068*	-0.017
Macropharyngodon meleagris	-0.066***	0.042	0.0057**	-0.00018
Macropharyngodon ornatus	0.12***	-0.82***	0.00085	-0.08**

Species	DHW	SSTA	Coral Loss	MHW Gradient
Meiacanthus atrodorsalis	0.037***	-0.38***	0.0065***	-0.014
Meiacanthus ditrema	0.032*	-0.23	-0.00042	-0.044
Meiacanthus grammistes	0.0072	-0.13	0.011***	0.042*
Meiacanthus lineatus	0.05**	-0.44**	0.0031	-0.039
Meiacanthus phaeus	-0.042*	-0.025	-0.0051*	-0.014*
Melichthys niger	-0.031*	0.083	-0.0058*	-0.062
Melichthys vidua	-0.012	0.18	-0.0049*	-0.018
Monotaxis grandoculis	0.016*	-0.19*	-0.0031*	-0.096*
Monotaxis heterodon	-0.059***	0.15	0.0066***	0.056*
Mulloidichthys flavolineatus	0.0053	0.043	-0.0011	-0.002
Mulloidichthys vanicolensis	-0.0019	-0.12	-0.0019	-0.025
Myripristis adusta	0.042**	-0.19	-0.0082***	-0.074*
Myripristis berndti	-0.024**	-0.027	-0.001	-0.07
Myripristis hexagona	0.015	-0.1	-0.0037	-0.033
Myripristis kuntee	-0.032**	0.12	0.001	0.0085
Myripristis murdjan	0.023*	-0.41***	-0.0054**	-0.26***
Myripristis violacea	0.032***	0.089	-0.013***	-0.11*
Myripristis vittata	0.03*	-0.035	-0.006*	-0.025
Naso annulatus	0.058***	-0.27*	-0.0011	-0.024
Naso brachycentron	0.0059	-0.2	-0.0059*	-0.1**
Naso brevirostris	-0.027**	0.032	0.00031	-0.034
Naso caesius	0.0047	0.14	-0.00078	0.013
Naso hexacanthus	0.055***	-0.46***	-0.0098***	-0.22***
Naso lituratus	-0.039***	0.2*	0.0017	0.067
Naso tonganus	0.046***	-0.56***	-0.005**	-0.26***
Naso unicornis	-0.036***	0.34***	0.0028*	0.13***
Naso vlamingii	0.051***	-0.12	-0.0049*	-0.058
Nemateleotris magnifica	0.025*	-0.28*	-0.0047*	-0.083**
Neoglyphidodon melas	0.019*	-0.14	-0.012***	-0.19***
Neoglyphidodon nigroris	0.029*	-0.15	-0.005*	-0.061*
Neoniphon argenteus	0.049**	-0.094	-0.0087***	-0.038
Neoniphon opercularis	0.077***	-0.47***	0.0048*	-0.018
Neoniphon sammara	0.072***	-0.47***	-0.0085***	-0.12***
Neopomacentrus azysron	-0.019	0.072	-0.0083***	-0.12**

Species	DHW	SSTA	Coral Loss	MHW Gradient
Neopomacentrus bankieri	-0.017	-0.21	-0.0089**	-0.24***
Neopomacentrus cyanomos	0.0068	-0.28*	0.0034	-0.042
Novaculichthys taeniourus	-0.0031	-0.056	-0.002	-0.023
Odonus niger	0.038*	-0.24	0.00046	-0.025
Ogilbyina queenslandiae	0.016	-0.34*	0.0052*	-0.023
Ostorhinchus nigrofasciatus	-0.026*	0.28*	0.0072**	0.072**
Ostorhinchus properuptus	-0.0037	-0.66***	0.0014	-0.19***
Ostracion cubicus	-0.047***	0.29**	0.0032*	0.025*
Ostracion meleagris	0.00043	0.0074	0.0019	0.00058
Oxycheilinus digrammus	-0.0062	-0.17*	0.0018	-0.066
Oxycheilinus orientalis	0.048***	-0.21*	0.0028	0.0066
Oxycheilinus unifasciatus	-0.0064	-0.14	0.0082***	0.059
Oxymonacanthus longirostris	-0.02*	-0.38***	-0.01***	-0.36***
Paracirrhites arcatus	0.005	-0.027	-0.0057**	-0.052*
Paracirrhites forsteri	0.038***	-0.13	-0.0054**	-0.086*
Paraluteres prionurus	0.028*	-0.32**	-0.0011	-0.034*
Parapercis australis	-0.017	-0.1	0.011***	0.018*
Parapercis clathrata	0.02*	-0.37**	0.0027	-0.063
Parapercis millepunctata	0.0089	-0.061	-0.00048	-0.023
Parapercis pacifica	0.039*	-0.18	-0.000041	-0.017
Parupeneus barberinoides	0.0056	-0.11	0.0013	-0.026
Parupeneus barberinus	-0.0042	-0.2*	-0.000027	-0.095*
Parupeneus ciliatus	0.01	-0.29*	-0.0049*	-0.11**
Parupeneus crassilabris	0.031**	-0.1	0.0043*	0.038
Parupeneus cyclostomus	0.0058	0.074	-0.0033*	-0.023
Parupeneus multifasciatus	0.036***	-0.41***	0.0042**	-0.082
Parupeneus pleurostigma	-0.021*	-0.022	0.0039*	-0.0025
Pempheris oualensis	-0.026*	-0.33**	-0.002	-0.083***
Pentapodus aureofasciatus	0.027*	-0.28*	0.007*	-0.0034
Pervagor alternans	-0.041*	-0.1	0.00066	-0.031
Pervagor janthinosoma	-0.032*	0.064	0.0018	-0.0098
Pictichromis paccagnellae	-0.016	-0.17	-0.004	-0.18*
Plagiotremus laudandus	0.016	-0.26*	-0.0027	-0.055*
Plagiotremus rhinorhynchos	0.01	0.094	0.0044*	0.14**

Species	DHW	SSTA	Coral Loss	MHW Gradient
Plagiotremus tapeinosoma	-0.0075	-0.24**	0.0022	-0.11*
Platax pinnatus	0.009	-0.026	-0.0033	-0.026
Platax teira	0.018	-0.38**	-0.0018	-0.17**
Plectorhinchus chaetodonoides	-0.0021	0.07	-0.00058	0.0049
Plectorhinchus lessonii	-0.02	0.038	-0.0025	-0.068
Plectorhinchus lineatus	0.03*	-0.044	-0.0057*	-0.031
Plectorhinchus multivittatus	0.0013	-0.0013	-0.0016	-0.034
Plectorhinchus picus	0.0081	-0.26*	0.0048*	-0.018
Plectorhinchus vittatus	0.1***	-0.65***	0.0018	-0.064
Plectroglyphidodon dickii	-0.003	0.19*	-0.013***	-0.054**
Plectroglyphidodon johnstonianus	-0.014	0.24*	-0.017***	-0.061***
Plectroglyphidodon lacrymatus	0.05***	0.057	0.0026	0.1**
Plectropomus areolatus	0.0041	0.4**	0.00023	0.048**
Plectropomus laevis	-0.043***	0.4***	0.004**	0.068***
Plectropomus leopardus	-0.011	-0.27**	-0.0061***	-0.29***
Plectropomus maculatus	-0.03*	-0.22*	0.000048	-0.099*
Plotosus lineatus	-0.014	-0.1	0.0024	-0.0058
Pomacanthus imperator	0.0011	-0.028	-0.00036	-0.015
Pomacanthus navarchus	0.0043	-0.0073	-0.0012	-0.025
Pomacanthus semicirculatus	-0.026	-0.14	0.00041	-0.054
Pomacanthus sexstriatus	0.041***	-0.53***	0.004*	-0.11*
Pomacentrus adelus	0.051***	-0.2	0.0029	0.018
Pomacentrus alexanderae	0.037*	-0.35*	0.0012	-0.01
Pomacentrus amboinensis	0.055***	-0.64***	0.0034*	-0.13**
Pomacentrus australis	-0.033*	-0.19	-0.0012	-0.075*
Pomacentrus bankanensis	-0.051***	0.1	0.004*	0.013
Pomacentrus brachialis	-0.027*	0.048	0.0055*	0.041
Pomacentrus chrysurus	0.024*	-0.1	0.01***	0.024**
Pomacentrus coelestis	-0.043***	-0.16*	0.0039*	-0.075
Pomacentrus grammorhynchus	-0.026*	-0.3**	-0.0051*	-0.17***
Pomacentrus imitator	-0.023	-0.76***	0.0059*	-0.16**
Pomacentrus lepidogenys	0.018	-0.28**	-0.0034*	-0.1**
Pomacentrus milleri	-0.017	0.059	-0.00003	-0.02
Pomacentrus moluccensis	-0.058***	0.38***	-0.01***	-0.064

Species	DHW	SSTA	Coral Loss	MHW Gradient
Pomacentrus pavo	-0.027*	0.23*	0.0022	0.019
Pomacentrus philippinus	-0.0036	-0.027	0.0037*	0.012
Pomacentrus vaiuli	-0.049***	0.65***	0.002	0.2***
Pomacentrus wardi	0.023	-0.39**	0.007**	-0.026
Pomachromis richardsoni	-0.039**	-0.11	0.0013	-0.049
Premnas biaculeatus	-0.012	-0.25*	-0.0023	-0.075**
Priacanthus hamrur	0.011	-0.083	-0.00082	-0.016
Pristiapogon kallopterus	0.011	-0.37**	0.0063*	-0.042
Psammoperca waigiensis	-0.022	0.17	-0.0017	-0.0054
Pseudanthias dispar	0.042*	-0.26	-0.011***	-0.072**
Pseudanthias pascalus	-0.012	-0.23	-0.0024	-0.071*
Pseudanthias squamipinnis	0.017	-0.2	-0.0058*	-0.095*
Pseudanthias tuka	0.016	0.13	-0.000027	0.028
Pseudobalistes flavimarginatus	0.017	-0.28*	0.0059*	-0.011
Pseudocheilinus evanidus	0.025*	-0.36**	0.000032	-0.1*
Pseudocheilinus hexataenia	0.022**	-0.18*	-0.013***	-0.4***
Pseudocheilinus octotaenia	0.013	-0.46***	0.0016	-0.09*
Pseudochromis fuscus	-0.0023	0.22**	-0.0023	0.041
Pseudodax moluccanus	0.034*	-0.33*	-0.0043	-0.071*
Pseudolabrus guentheri	-0.026	-0.49***	0.0016	-0.096**
Ptereleotris evides	-0.0099	0.027	0.0094***	0.19***
Pterocaesio digramma	-0.022*	-0.016	-0.004*	-0.12*
Pterocaesio marri	0.029*	-0.28*	-0.00019	-0.056
Pterocaesio tile	0.042***	-0.13	-0.0018	-0.025
Pterocaesio trilineata	0.034**	-0.16	-0.0072**	-0.069*
Pygoplites diacanthus	0.023**	-0.21*	-0.006***	-0.16***
Rhabdamia gracilis	0.019	-0.53***	0.0042	-0.11*
Rhinecanthus aculeatus	0.016	-0.16	0.0039	-0.0065
Rhinecanthus rectangulus	0.012	-0.23	0.0051*	-0.015
Salarias fasciatus	-0.021	0.027	0.0063*	0.0031
Sargocentron caudimaculatum	0.035***	-0.26*	0.0025	-0.029
Sargocentron diadema	-0.0048	0.024	0.006*	0.006
Sargocentron rubrum	-0.0096	-0.19	-0.0021	-0.098*
Sargocentron spiniferum	0.044***	-0.33***	-0.01***	-0.36***

Species	DHW	SSTA	Coral Loss	MHW Gradient
Saurida nebulosa	0.00018	-0.13	-0.0012	-0.055
Scarus altipinnis	-0.029**	-0.077	0.0061***	0.0026
Scarus chameleon	-0.049***	0.19*	-0.0022	-0.013
Scarus dimidiatus	-0.015	0.016	-0.0014	-0.051
Scarus flavipectoralis	0.014	-0.34***	-0.0042*	-0.23***
Scarus forsteni	0.0065	0.012	0.0076***	0.14**
Scarus frenatus	-0.033***	0.2*	-0.0053***	-0.069
Scarus ghobban	-0.012	0.15	0.00083	0.028
Scarus globiceps	-0.031**	0.071	-0.0059**	-0.07*
Scarus longipinnis	-0.026*	-0.05	0.013***	0.012*
Scarus niger	0.0011	-0.074	-0.0029*	-0.099*
Scarus oviceps	-0.098***	0.7***	-0.0021	0.0055*
Scarus prasiognathos	-0.029*	0.45***	-0.0087***	-0.0097
Scarus psittacus	-0.033**	0.021	-0.0002	-0.027
Scarus rivulatus	-0.017	0.05	0.001	0.0043
Scarus rubroviolaceus	0.025*	-0.66***	0.0054*	-0.19**
Scarus schlegeli	-0.027***	0.14*	0.0089***	0.23***
Scarus spinus	-0.027*	0.046	0.0022	-0.007
Scolopsis bilineata	-0.068***	0.48***	0.0033*	0.2***
Scolopsis margaritifer	0.037**	-0.61***	0.00026	-0.15***
Scolopsis monogramma	0.053***	-0.39**	0.0088**	0.013
Scolopsis trilineata	0.0039	-0.089	0.0022	-0.007
Scomberoides lysan	0.033*	-0.15	-0.0035	-0.039
Serranocirrhitus latus	0.016	-0.25	0.00031	-0.028
Siganus argenteus	0.036**	-0.42***	0.008**	-0.019
Siganus corallinus	-0.044***	0.45***	-0.0058**	0.018
Siganus doliatus	-0.06***	0.31**	0.0036	0.039*
Siganus fuscescens	-0.035*	0.27*	-0.002	-0.0029
Siganus lineatus	-0.0064	0.11	0.005*	0.029*
Siganus puellus	0.0023	0.23*	-0.00061	0.02*
Siganus punctatissimus	0.026	-0.043	-0.0012	-0.012
Siganus punctatus	-0.022*	-0.16	0.0025	-0.09
Siganus virgatus	-0.021	0.11	0.0065*	0.014*
Siganus vulpinus	-0.043***	0.37***	-0.0063**	-0.012

Species	DHW	SSTA	Coral Loss	MHW Gradient
Signigobius biocellatus	0.043*	-0.55***	-0.003	-0.074***
Stegastes apicalis	-0.012	-0.2	0.0024	-0.043
Stegastes fasciolatus	-0.028***	0.23**	0.0043**	0.068**
Stegastes gascoynei	-0.08***	0.13	0.0033	-0.00078
Stegastes nigricans	0.038***	0.059	-0.0019	0.029
Stegastes obreptus	-0.028	0.19	0.0011	0.0053
Stegastes punctatus	-0.00054	-0.065	0.00048	-0.021
Stethojulis bandanensis	-0.0093	-0.018	0.0091***	0.16***
Stethojulis interrupta	0.0016	-0.43***	0.0013	-0.21**
Stethojulis strigiventer	-0.033**	0.53***	0.00081	0.1***
Sufflamen bursa	-0.015	0.083	0.0017	0.0055
Sufflamen chrysopterum	0.027**	-0.23*	0.0095***	0.073*
Symphorichthys spilurus	0.0095	0.018	-0.00054	-0.012
Synodus binotatus	0.0064	-0.22*	0.0036	-0.034
Synodus dermatogenys	0.0012	-0.39**	0.0063*	-0.059
Synodus jaculum	0.019	-0.22	-0.0039	-0.07*
Synodus variegatus	-0.055***	-0.12	0.0025	-0.14*
Thalassoma amblycephalum	-0.003	-0.14	0.00055	-0.066
Thalassoma hardwicke	-0.031***	0.48***	-0.0053***	0.068*
Thalassoma jansenii	0.039**	-0.62***	0.00084	-0.18***
Thalassoma lunare	0.0088	-0.35***	-0.0031*	-0.19***
Thalassoma lutescens	-0.057***	0.42***	-0.0036*	0.025
Thalassoma nigrofasciatum	-0.017	-0.066	-0.00084	-0.055
Thalassoma quinquevittatum	-0.009	0.13	0.0093***	0.067***
Triaenodon obesus	-0.027**	-0.23**	-0.0029*	-0.24***
Valenciennea longipinnis	0.01	-0.28*	0.0044	-0.025
Valenciennea strigata	0.017	-0.22*	0.007**	0.006
Variola louti	-0.025*	0.016	0.00038	-0.016
Zanclus cornutus	0.012*	0.17*	0.0013	0.15**
Zebrasoma scopas	-0.083***	0.57***	-0.023***	-0.25***

Table S2: Trait groupings for cluster analysis.

Trait	Low values	Medium values	High values
SGI	< 15	15 <= SGI < 22	>= 22
Maximum Length (cm)	<= 12	12 < ML < 30	>= 30
Thermal Midpoint (^{o}C)	< 24.8	24.8 <= TM < 25.3	>= 25.3

Table S3: Synchronicity of species-level responses to coral cover, sea surface temperature anomaly (SSTa) and degree heating weeks (DHW).

Compared variables	Both negative (n)	Both positive (n)	Negative-positive (n)	Positive-negative (n)
Coral-DHW	20	3	7	3
Coral-SSTa	7	0	12	2
DHW-SSTa	1	0	25	38
